# Towards planetary boundary sustainability of food processing wastewater, by resource recovery & emission reduction: A process system engineering perspective^☆^

**DOI:** 10.1016/j.ccst.2024.100319

**Published:** 2024-12

**Authors:** Alex Durkin, Tom Vinestock, Miao Guo

**Affiliations:** a Department of Chemical Engineering, Imperial College London, SW7 2AZ, UK; b Department of Engineering, King’s College London, WC2R 2LS, UK

**Keywords:** Resource recovery, Surrogate modelling, Process synthesis, Planetary boundaries, Wastewater treatment, Carbon emissions, Nitrogen emissions, Biorefinery, Process systems engineering, Food & drink processing

## Abstract

Meeting the needs of a growing population calls for a change from linear production systems that exacerbate the depletion of finite natural resources and the emission of environmental pollutants. These linear production systems have resulted in the human-driven perturbation of the Earth’s natural biogeochemical cycles and the transgression of environmentally safe operating limits. One solution that can help alleviate the environmental issues associated both with resource stress and harmful emissions is resource recovery from waste. In this review, we address the recovery of resources from food and beverage processing wastewater (FPWW), which offers a synergistic solution to some of the environmental issues with traditional food production. Research on resource recovery from FPWW typically focuses on technologies to recover specific resources without considering integrative process systems to recover multiple resources while simultaneously satisfying regulations on final effluent quality. Process Systems Engineering (PSE) offers methodologies able to address this holistic process design problem, including modelling the trade-offs between competing objectives. Optimisation of FPWW treatment and resource recovery has significant scope to reduce the environmental impacts of food production systems. There is significant potential to recover carbon, nitrogen, and phosphorus resources while respecting effluent quality limits, even when the significant uncertainties inherent to wastewater systems are considered. This review article gives an overview of the environmental challenges we face, discussed within the framework of the planetary boundary, and highlights the impacts caused by the agri-food sector. This paper also presents a comprehensive review of the characteristics of FPWW and available technologies to recover carbon and nutrient resources from wastewater streams with a particular focus on bioprocesses. PSE research and modelling advances are discussed in this review. Based on this discussion, we conclude the article with future research directions.

## 1 Introduction to planetary boundaries

### 1.1 Limits to growth

Exponential increases in global population and economic output, coupled with environmentally damaging modalities of production and consumption, are driving climate change and risk tipping the Earth system from the stable Holocene into an unknown, less hospitable Anthropocene (Rockstrom et al., 2009). This destabilisation of the planet is driven by human activities founded in the linear “take-make-waste” economy upon which modern socio-economic development is based. The linear take-make-waste economy generates large amounts of waste and increases resource scarcity as growing demand is met by increasing consumption of raw materials and energy. With the world’s urban population expected to rise by 2.5 billion by 2050 (United Nations, 2018), and emerging markets characterised by growing consumption demands, the environmental problems associated with a linear economy are becoming evermore marked (Clark et al., 2016).

Supporting an increasing global population with improving standards of living demands increased water, energy, and food (WEF) resources. In a linear economy, this growth is accompanied by an increase in the production of waste and environmentally harmful emissions. Therefore, there exists an important challenge in ensuring WEF resource security, that requires delicately balancing increases in production with a reduction in wasteful and environmentally-damaging practices. An additional difficulty in ensuring WEF resource security lies in the complex interdependencies between the individual resource systems; food, water, energy, and transport are closely linked and all compete for Earth’s finite resources (Food and Agriculture Organization, 2014).

**Fig. 1:**
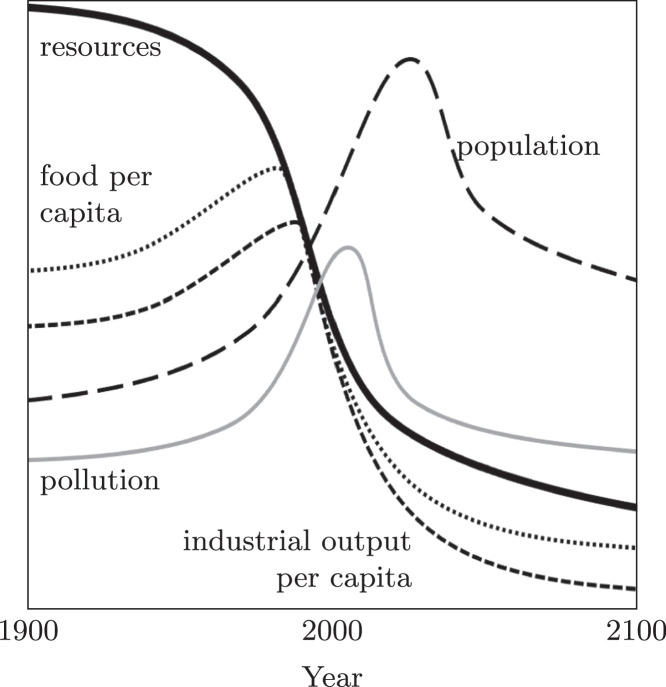
The World Model. Adapted from Meadows et al. (1972). Note that the vertical scales are omitted deliberately to emphasise the general behavior predicted by the model, rather than the numerical values, which are approximate.

The challenge of sustainably supporting socio-economic growth has long been a topic of discussion. In 1972, *Limits to Growth* (Meadows et al., 1972) was published in which the authors declared that anthropogenic systems would result in a destabilisation of the Earth system. In *Limits to Growth*, the authors presented the “World Model” in which the availability of Earth’s natural resources constrain global populations, industrial output, and food production. It argued that exponential population increase, and the necessary food production and industrial output to support it, cannot be sustained by a linear resource-to-waste system. The result of continued growth in this economic model was projected to be a peak in pollution followed shortly by a peak in global population caused by the erosion of the capacity of critical Earth systems (Fig. 1).

Whilst the World Model has received criticism for its simplicity and its perceived pessimism (Cole and Freeman, 1973), it has been largely accepted that major changes to the linear economy are essential to avoid a peak in economic development by 2040 (Herrington, 2021). Other studies have validated the world model against real data over a 30 year period, with comparable results (Turner, 2008).

### 1.2 Planetary boundaries

The planetary boundaries (PBs) framework was introduced to quantify the safe operating limits on nine Earth system processes: climate change, land use change, human intervention in nutrient (nitrogen and phosphorus) cycles, freshwater consumption, ocean acidification, ozone depletion, biodiversity loss, chemical pollution, and atmospheric particulate loading (Rockstrom, Steffen, Noone, Persson, Chapin, Lambin, Lenton, Scheffer, Folke, Schellnhuber, Nykvist, de Wit, Hughes, van der Leeuw, Rodhe, Sörlin, Snyder, Costanza, Svedin, Falkenmark, Karlberg, Corell, Fabry, Hansen, Walker, Liverman, Richardson, Crutzen, Foley, 2009, Steffen, Richardson, Rockström, Cornell, Fetzer, Bennett, Biggs, Carpenter, de Vries, de Wit, Folke, Gerten, Heinke, Mace, Persson, Ramanathan, Reyers, Sörlin, 2015). Akin to the tipping point in *Limits to Growth*, the PBs defined quantified thresholds beyond which the Earth system behaviour is unprecedented (Rockstrom et al., 2009). Specifically, the PBs framework highlights a metric or control variable for each of the nine Earth subsystems. Each control variable was assigned a threshold that defined the PB in addition to a zone of uncertainty around each PB. As a result it is possible to qualitatively categorise subsystems as either: below the boundary (safe); in a zone of uncertainty with increasing risk; or beyond the zone of uncertainty and high risk (Steffen et al., 2015). The latest assessments determined that the safe limits for biodiversity loss and human intervention in the nitrogen (N) and phosphorus (P) cycles have already been transgressed and are therefore high risk (Steffen et al., 2015). Studies also place climate change, land use change, and freshwater consumption in a zone of uncertainty with elevated risk (Gerten, Hoff, Rockström, Jägermeyr, Kummu, Pastor, 2013, Steffen, Richardson, Rockström, Cornell, Fetzer, Bennett, Biggs, Carpenter, de Vries, de Wit, Folke, Gerten, Heinke, Mace, Persson, Ramanathan, Reyers, Sörlin, 2015). While agriculture and industry are important for advancing human development, modern farming practices and resource exploitation are major threats to the PBs (Keijer, Bakker, Slootweg, 2019, Rockström, Edenhofer, Gaertner, DeClerck, 2020, Sutton, Oenema, Erisman, Leip, van Grinsven, Winiwarter, 2011). These studies reaffirm the link between wastefulness and growing environmental concerns, and emphasise the challenge of sustainably supporting global population increase and continued socio-economic development.

A challenge associated with the PB framework is the selection of control variables for each Earth system process, as the proxy measures selected could be unrepresentative of the wider underlying problems they are supposed to represent. As a result, it is possible that PBs could be transgressed without significant consequence. Equally, it is possible that irreversible planetary change could occur even if none of the PBs are transgressed. Furthermore, while different PBs relate to different processes, they are closely linked and should not be addressed in isolation from one another (Rockstrom et al., 2009). Another challenge pertains to the quantification of each PB threshold with the authors admitting that some PBs are merely their best guesses, resulting in large zones of uncertainty (Rockstrom et al., 2009). Only three of the nine PBs are intrinsically global in scale, the other six being intrinsically local or regional, albeit possible to aggregate globally (Rockström et al., 2009). However, because of this global aggregation, there exists a challenge in translating the global PBs into metrics for regional or local scales. Finally, there is a similar challenge in allocating PB budgets between individual activities within the economy (Clift et al., 2017). To operationalise the PBs in this way, life cycle assessment (LCA), has been highlighted as a complementary method to allocate available PB operating budgets, resulting in the concept of PB-LCA (Algunaibet, Pozo, Galán-Martin, Huijbregts, Mac Dowell, Guillén-Gosálbez, 2019, Bunsen, Berger, Finkbeiner, 2021, Lima, Ferrer, Kjerstadius, Ryberg, McConville, 2023, Tulus, Pérez-Ramírez, Guillén-Gosálbez, 2021). Further discussion and criticism of the PB framework can be found in de Vries et al. (2013).

Despite the shortcomings of PBs, they nonetheless provide quantified science-based targets within which humanity should aim to contain its activity to avoid destabilising key Earth systems. Table 1 shows the PBs, zones of uncertainty, and current status of the control variables for 4 Earth systems of particular interest in this work. Specifically, climate change, human intervention in the N and P cycles, freshwater use, and land use change are considered due to their particular relevance to the WEF nexus.

**Table 1:** Planetary boundaries for climate change, land-use change, freshwater consumption, and human intervention in the nitrogen and phosphorus cycles. The lower or conservative threshold of the zone of uncertainty is indicated first; the upper or optimistic threshold is then indicated in brackets. Climate change control variable: atmospheric CO${}_{2}$ concentration, land-use change control variable: area of forested land as % of original forest cover, freshwater use control variable: consumptive blue water use, nitrogen (N) fixation control variable: industrial and intentional biological N fixation, phosphorus (P) intervention control variable: P flow from freshwater to ocean. Data source: (Richardson et al., 2023) except for freshwater consumption data, from Steffen et al. (2015).

Earth system	PB (zone of uncertainty)	Current status
Climate change - CO${}^{2}$ Concentration	350 (450) ppm CO${}_{2}$	417 ppm CO${}_{2}$
Climate change - Radiative Forcing	+1.0 (1.5) W m${}^{-2}$	2.91 W m${}^{-2}$
N fixation	62 (82) Mt N y${}^{-1}$	190 Mt N y${}^{-1}$
P loading	11 (100) Mt P y${}^{-1}$	22.6 Mt P y${}^{-1}$
Freshwater consumption	4000 (6,000) km${}^{3}$ y${}^{-1}$	2,600 km${}^{3}$ y${}^{-1}$
Land use change	75 (54) %	60%

#### 1.2.1 Climate change

The most widely acknowledged environmental concern of today is climate change, which has been assigned two PB control variables: the concentration of carbon dioxide (CO${}_{2}$) in the atmosphere and the increase in radiative forcing compared to preindustrial levels (Rockstrom et al., 2009). The boundaries established for the two variables are 350 ppm and 1.0 W$m^{-2}$, respectively, (Rockstrom et al., 2009). Radiative forcing is the more comprehensive measure, accounting for other greenhouse gas (GHG) emissions, aerosols, and other anthropogenic impacts on the global energy balance. However, the atmospheric CO${}_{2}$ concentration is an important control variable due to the large volume of anthropogenic emissions and its long half-life in the atmosphere (Steffen et al., 2015). Current atmospheric CO${}_{2}$ concentration is estimated to be 417 ppm (Richardson et al., 2023). The increase in CO${}_{2}$ concentration has been driven by fossil fuels combustion and land use change, which move carbon from natural reserves, such as soil and subterranean fossil fuel deposits, to the atmosphere (Olah et al., 2011). These impacts are exacerbated by climate change itself, with rising global temperatures increasing the emission of CO${}_{2}$ from land and ocean carbon sinks (forests and phytoplankton). Historically, forests and phytoplankton have each been responsible for removing about 25% of anthropogenic emissions from the atmosphere (Friedlingstein, 2015).

Estimates of the climate change PB and control variables vary widely depending on which anthropogenic activities (primarily the inclusion/exclusion of emissions attributed to land use change) and GHGs are included. Some studies have operationalised the climate change PB by explicitly specifying safe operating budgets in terms of anthropogenic GHG emissions. To remain in accordance with the Paris Agreement to keep mean global temperatures within 1.5–2 ${}^{\circ }$C of pre-industrial temperatures, remaining carbon budgets (between 2011 and 2100) of 800 Gt CO${}_{2}$ or 1000 Gt CO${}_{2}$e including CO${}_{2}$, methane (CH${}_{4}$), and nitrous oxide (N${}_{2}$O) have been determined (Willett et al., 2019). In addition to CO${}_{2}$ which accounted for 74.4% of global carbon dioxide equivalent (CO${}_{2}$e) emissions in 2016, the remaining 25.6% was attributed to other GHGs, with 17.3% and 6.2% of GHG emissions from CH${}_{4}$ and N${}_{2}$O, respectively, and with the remaining 2.1% of GHG emissions attributed to fluoride gases, not accounting for emissions due to land use change (Ritchie et al., 2020c). When land use change is accounted for, GHG emissions in 2019 were over 50 Gt CO${}_{2}$e, with about 42 Gt CO${}_{2}$e (∼80%) emitted as CO${}_{2}$ (Ritchie et al., 2020c).

**Fig. 2:**
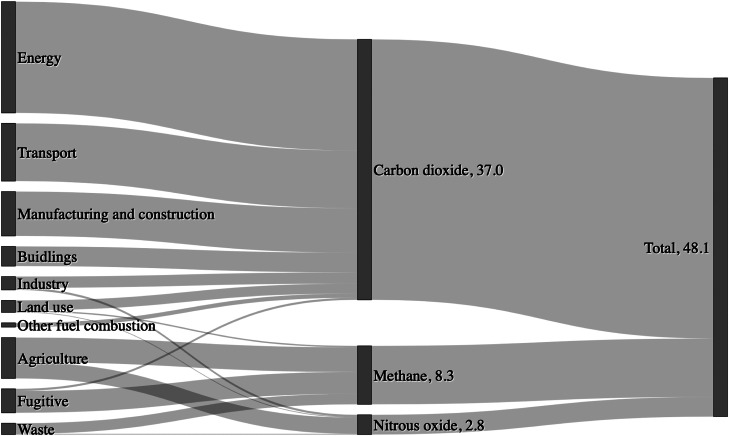
Sources of global greenhouse gas emissions (not including fluoride gases) in 2019. Units are in Gt CO${}_{2}$e y${}^{-1}$. Data source: (Ritchie et al., 2020c).

Fig. 2 shows the contribution of different sectors to global emissions of CO${}_{2}$, CH${}_{4}$, and N${}_{2}$O. Total emissions of these GHGs were 48.1 Gt CO${}_{2}$e with 77%, 17% and 6% from CO${}_{2}$, CH${}_{4}$, and N${}_{2}$O, respectively. Energy production (15.8 Gt CO${}_{2}$), transport (8.2 Gt CO${}_{2}$), and manufacturing and construction (6.3 Gt CO${}_{2}$) contribute greatly to CO${}_{2}$ emissions primarily by the combustion of fossil fuels. In fact, fossil fuels contribute at least 90% of total CO${}_{2}$ emissions with about 95% of fossil fuel emissions attributed to coal, oil, and natural gas, each emitting 41%, 32%, and 22%, respectively. CO${}_{2}$ emissions from gas accounted for 22% of fossil fuel emissions (with the remaining 5% attributable to industry (primarily cement production) using a mixture of fossil fuels) (Ritchie et al., 2020c).

Whilst the combustion of fossil fuels dominates CO${}_{2}$ emissions, the emission of more potent CH${}_{4}$ and N${}_{2}$O GHGs is dominated by agriculture (3.5 Gt CO${}_{2}$e and 2.3 Gt CO${}_{2}$e, respectively). Specifically, CH${}_{4}$ and N${}_{2}$O have global warming potentials (over a 100 year timescale and not accounting for climate feedback) of 28 and 265 times that of CO${}_{2}$, respectively. However, the short atmospheric lifetime of CH${}_{4}$ compared to CO${}_{2}$ (decades compared to centuries) means that tackling CH${}_{4}$ emissions is an effective way to rapidly mitigate a fraction of the transgression of the climate change PB (Shindell et al., 2012). The majority of agricultural CH${}_{4}$ emissions come from enteric fermentation in livestock and from rice production. Agricultural N${}_{2}$O emissions come primarily from synthetic and organic (manure) fertilisers. In addition to agriculture, a significant amount of CH${}_{4}$ emissions (3.1 Gt CO${}_{2}$e) come from fugitive emissions such as leaks from gas pipelines. Organic waste also decomposes in landfills emitting large amounts of CH${}_{4}$ (1.5 Gt CO${}_{2}$e) and smaller amounts of N${}_{2}$O (Ritchie et al., 2020c).

In summary, transgression of the climate change PB is driven by the combustion of fossil fuels (contributed to 90% of CO${}_{2}$ emissions) and agriculture. Overall, it is estimated that the food system, including land use change, farming practices, processing, transport, packaging, and retail, is responsible for around one quarter of global GHG emissions (Campbell, Beare, Bennett, Hall-Spencer, Ingram, Jaramillo, Ortiz, Ramankutty, Sayer, Shindell, 2017, Poore, Nemecek, 2018). A dramatic overhaul of the energy and food production systems is therefore vital if the values of the climate change PB indicators are to be stabilised or reduced.

#### 1.2.2 Nitrogen fixation

The natural nitrogen cycle is being destabilised by human activities, primarily use of the Haber-Bosch process, which transforms the inert nitrogen gas (N${}_{2}$), that makes up 78% of the atmosphere, into the more reactive compound ammonia (NH${}_{3}$) (Sutton et al., 2011). Since 1913, the Haber-Bosch process has enabled increased production of nitrogen-containing feedstocks for use in industry such as nitric acid, explosives such as trinitrotoluene (TNT), and cyanide, a nylon precursor. Most importantly, however, the industrial fixation of nitrogen enables the production of large amounts of fertiliser to feed growing global populations (Lehnert et al., 2018).

The Haber-Bosch process reacts nitrogen, distilled from liquefied air, with hydrogen generated from methane and steam reforming to produce ammonia over an iron catalyst (Reaction RR1). The high temperatures and pressures (about $500\;{}^{\circ }$C and 200 Bar) required by the Haber-Bosch process needs large amounts of energy, typically provided by fossil fuels (Smith et al., 2020). Due to the scale of Haber-Bosch ammonia production, this process accounts for 1–2% of all fossil fuel consumption and about 1.2–1.4% of global CO${}_{2}$ emissions (Matassa et al., 2023).

(R1) $$N_{2}\left(g\right)+3H_{2}\left(g\right)⟶{\text{NH}}_{3}\left(g\right)$$

Nitrogen is required by all living organisms, existing biologically in nucleic acids, amino acids, vitamins, cofactors and hormones. Nitrogen is therefore fundamental to feeding the worlds 8 billion people: without the Haber-Bosch process to provide nitrogen-rich fertilisers, it is estimated that 30–50% of the worlds harvest would be lost (Erisman et al., 2008). In a similar way to fossil fuels, which have revolutionised life on Earth by providing a cheap and convenient source of energy, Haber-Bosch, and the cheap fertilisers it enables, have revolutionised human life by supporting production of cheap and plentiful food. However, as with carbon, this large-scale human interference in the nitrogen cycle is a double-edged sword; it is also causing great environmental damage.

The Haber-Bosch process is not the only pathway for fixing nitrogen. For billions of years, bacteria have been fixing nitrogen from the atmosphere. However, this biological pathway is extremely energy inefficient. Breaking the triple N-N bond in N${}_{2}$, whether chemically or biologically, requires a large amount of energy. While the Haber-Bosch process uses high pressure and temperatures to break these bonds, the biological pathway uses an enzyme. Specifically, the nitrogenase enzyme catalyses the 6e${}^{-}$ reduction of N${}_{2}$ to NH${}_{3}$ (Lehnert et al., 2018). Although only 6 electrons are required theoretically, the nitrogenase mechanism including adenosine triphophate (ATP) hydrolysis necessitates the formation of a wasteful 1 mole of H${}_{2}$ per mole of N${}_{2}$ reduced, resulting in consumption of 2 extra electrons and 4 additional ATP per N${}_{2}$ (Hoffman et al., 2014).

(R2) $$\begin{array}{ccc} & & N_{2}+16\text{ATP}+16H_{2}O+8e^{-}+8H^{+}\\ & & \;⟶\\ & & 2{\text{NH}}_{3}+H_{2}+16\text{ADP}+16P_{i} \end{array}$$

**Fig. 3:**
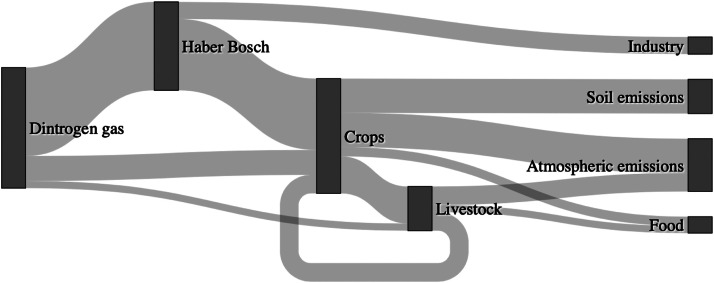
Nitrogen (N) flows due to industrial nitrogen fixation via the Haber Bosch process (124 Mt N y${}^{-1}$), intended biological fixation by crops (35 Mt N y${}^{-1}$), and deposition via livestock rearing (10 Mt N y${}^{-1}$). 24 Mt y${}^{-1}$ of the nitrogen fixed via the Haber Bosch process is used in industry whilst the rest is used to produce fertiliser (100 Mt N y${}^{-1}$). 52 Mt N y${}^{-1}$ of nitrogen in crops is consumed as feed for livestock wherein 26 Mt N y${}^{-1}$ of this is recycled during manure application. The storage of this manure also results in 26 Mt N y${}^{-1}$ of N emissions to the environment attributed to livestock. The remaining 10 Mt N y${}^{-1}$ N embedded in livestock is utilised as food for human consumption. 13 Mt N y${}^{-1}$ of crop-based N is consumed as food by humans whilst 48 Mt N y${}^{-1}$ is emitted as runoff to the soil and another 48 Mt N y${}^{-1}$ is emitted to the atmosphere via volatilisation. NB This figure neglects unintended NO${}_{x}$ emissions from combustion. Data sources: (Galloway, Townsend, Erisman, Bekunda, Cai, Freney, Martinelli, Seitzinger, Sutton, 2008, Matassa, Batstone, Hülsen, Schnoor, Verstraete, 2015, Smith, K.Hill, Torrente-Murciano, 2020).

Fig. 3 shows the flows of reactive nitrogen fixed via the Haber-Bosch or via intentional biological fixation. Only 14% of the natural dinitrogen gas fixed into reactive nitrogen is consumed by humans. Another 14% is embedded within other industrial non-food products with the remaining 72% wasted, either being emitted to the soil (28%) or to the atmosphere (44%). Nitrogen emitted to the soil can result in soil acidification (in the case of ammonium NH${}_{4}^{+}$) or runoff to water bodies causing increased aquatic toxicity or eutrophication. Nitrogen emissions to the atmosphere occurs as NH${}_{3}$, N${}_{2}$O or NO${}_{\text{x}}$. Only about 40% of the N applied to croplands is incorporated into the plant biomass whilst 30% is lost through gaseous volatilisation, and 30% from runoff (Matassa et al., 2015). However, exact quantification of these figures is complicated due to large uncertainties in the rates of biological N fixation, the amount of reactive N stored in environmental reservoirs, and the production rates of N${}_{2}$ by denitrification.

With regards to the PBs, nitrogen fixation was assigned the control variable of industrial and intentional biological fixation (driven by agricultural demand) of N${}_{2}$ to reactive nitrogen, with a boundary at 62 Mt N y${}^{-1}$ and a zone of uncertainty up to 82 Mt N y${}^{-1}$. This control variable value was estimated to be about 190 Mt N y${}^{-1}$, putting it beyond the zone of uncertainty and therefore in the high risk area (Table 1) (Richardson et al., 2023).

Total Haber-Bosch ammonia production is approximately 150 Mt y${}^{-1}$ (Smith et al., 2020) which is equivalent to 124 Mt y${}^{-1}$ of N fixation (a molar mass conversion factor of $\frac{14}{17}$) which is in good agreement with other estimates (Galloway, Townsend, Erisman, Bekunda, Cai, Freney, Martinelli, Seitzinger, Sutton, 2008, Gruber, Galloway, 2008). About 24 Mt y${}^{-1}$ (20%) of this fixated nitrogen is used in the industrial chemical production of explosives and polymers, whilst the majority (100 Mt y${}^{-1}$ or 80%) is utilised for fertilisers (Erisman, Sutton, Galloway, Klimont, Winiwarter, 2008, Galloway, Townsend, Erisman, Bekunda, Cai, Freney, Martinelli, Seitzinger, Sutton, 2008, Matassa, Batstone, Hülsen, Schnoor, Verstraete, 2015). These estimates neglect unintended NO${}_{\text{x}}$ emissions from fossil fuel combustion, which explains the discrepancy with the estimates of the current nitrogen PB control variable (190 Mt N y${}^{-1}$). Galloway et al. (2008) estimated total reactive N production including unintended NO${}_{\text{x}}$ emissions, and obtained a figure of 187 Mt y${}^{-1}$, albeit for 2005 (121 Mt y${}^{-1}$ from Haber-Bosch, 40 Mt y${}^{-1}$ from biological fixation, and 25 Mt y${}^{-1}$ from industrial NO${}_{\text{x}}$ emissions). However, there is high uncertainty in these values owing both to different categorisations of the N flows and the age of the data used; some of the data are almost 20 years old.

#### 1.2.3 Phosphorus loading

Human intervention in the phosphorus (P) cycle is the other half of the PB concerning anthropogenic impacts on biogeochemical flows Rockstrom et al. (2009). The conservative estimate for the PB of phosphorus flow from freshwater into the ocean is 11 Mt P y${}^{-1}$, with the zone of uncertainty extending to 100 Mt P y${}^{-1}$. As the lower limit is transgressed, an oceanic anoxic event becomes increasingly likely. The current control variable value was estimated to be 22 Mt P y${}^{-1}$, putting P loading in the zone of uncertainty with increasing risks (Steffen et al., 2015). There also exist regional boundaries for P loading, set to prevent eutrophication of freshwater. These local boundaries have already been widely transgressed in the same way as with the N fixation PB.

P is an essential element for supporting life on Earth. Specifically, it is used for energy generation during the conversion of ATP to adenosine diphophate (ADP) and phosphate. P also exists within deoxyribonucleic acid (DNA) or ribonucleic acid (RNA), playing a crucial role in the transfer of genetic information. P is therefore an essential nutrient within fertilisers which is the primary driver of the 148 Mt of finite phosphate rock mined annually. Specifically, between 90–96% of total anthropogenic P (23.5 Mt y${}^{-1}$) is added to cropland soils as fertiliser (Campbell et al., 2017), of which approximately 14 Mt y${}^{-1}$ (over half) is wasted as a pollutant (Ritchie et al., 2022b). Aside from fertiliser production, P is also used in the industrial production of flares, incendiary devices, and some detergents.

Only 15% of P entering the United States (U.S.) food supply chain is ingested by humans with 85% emitted as waste to the soil, manure, crop residues, or receiving water bodies via runoff or erosion (Suh, Yee, 2011, Yuan, Jiang, Sheng, Liu, Hua, Liu, Zhang, 2018). For P that exists within wastewater systems, less than 15% typically remains within the reclaimed water stream, between 10–20% is emitted to aquatic systems, and 70–80% is removed within via microbial uptake into solid organic waste for disposal (Qiao et al., 2011). Recent applications for this P-rich solid waste include further concentration of P into value-added fertiliser products (Margenot et al., 2019). Other options for reducing phosphorus loading include recovery of P from manure, municipal and food wastes, and reducing food waste (Campbell et al., 2017). Once again, the focus is on reducing the environmental impacts of agriculture as it has been estimated to account for over 90% of this PB (Campbell et al., 2017).

#### 1.2.4 Freshwater use

Another natural system being perturbed by anthropogenic activities is the water cycle. Specifically, the consumptive use of freshwater (where consumptive use is defined as conversion of water to a state which renders it unrecoverable from the immediate water environment) is causing water stress, with knock-on impacts on ecosystems. Freshwater runoff has decreased about 10% (estimates range from 5 to 15%) primarily due to withdrawals for agricultural irrigation (Haddeland et al., 2014).

The primary contributors to freshwater consumption are agricultural practices (including irrigation and rearing livestock), energy production, and industrial and domestic uses. Additionally, the conversion of freshwater (blue/green water) into wastewater (grey water) drives environmental problems such as water pollution and eutrophication. Drinking water and wastewater treatment systems account for 2% of energy use in the U.S, which is still dependent on fossil fuels, and so contribute not insignificantly to climate change (United States Environmental Protection Agency, 2022). Globally, these environmental problems will be exacerbated by increasing human development and the growing demands of urban populations. Such environmental problems (and political issues associated with water resource security) are increasing with climate change. Although, on a global scale, human impacts are relatively small, the greatest regional impacts are seen in large river basins and in water stressed regions such as Qatar, Israel, and Lebanon (Haddeland et al., 2014).

**Fig. 4:**
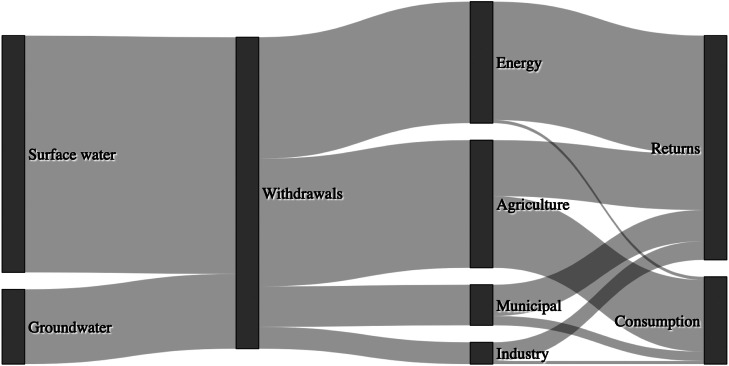
Freshwater use in the U.S. (adapted from Masters and Ela (2008)). 76% and 24% of freshwater withdrawals were from surface water and groundwater sources, respectively. Agriculture accounts for 41% of withdrawals while the energy sector (power plant cooling water), municipalities (public, domestic, and commercial use), and industry (including mining) account for 39%, 13%, and 7%, respectively. However, differing return rates mean that agriculture accounts for 86% of all consumptive use. Of total withdrawals, 72% are returned and 28% consumed.

Fig. 4 shows U.S. freshwater withdrawals and ultimate consumptive use by different sectors or returns to receiving water bodies (Masters and Ela, 2008). Specifically, 76% of freshwater withdrawals were from surface water with the remaining 24% from groundwater sources. The primary driver for U.S. freshwater withdrawals was agriculture, accounting for 41% of total withdrawals for irrigation and livestock. Agricultural purposes consumed 23% of the total withdrawals, accounting for 82% of total U.S. consumptive freshwater use. Despite the energy sector accounting for 39% of freshwater withdrawals for power plant cooling water, almost all of this (38% of total withdrawals) was returned to receiving water bodies, with the sector accounting for less than 4% of total consumptive use. The other sectors shown in Fig. 4 are municipalities including public, domestic, and commercial use, and industry which accounted for 13% and 7% of total U.S. freshwater withdrawals, respectively.

The PB for freshwater consumption was set at 4,000 km${}^{3}$ y${}^{-1}$ with a zone of uncertainty up to 6,000 km${}^{3}$ y${}^{-1}$ whilst the current state of the control variable is estimated to be 2,600 km${}^{3}$ y${}^{-1}$ although there is much uncertainty surrounding these estimates (Steffen et al., 2015). Agriculture has been highlighted as the primary driver of freshwater consumption, with irrigation accounting for 84% of consumptive freshwater use (Campbell et al., 2017). The growing global population and shift in dietary preference towards meat will demand even more water owing to the increased demand for water to grow crops as animal feed (the same is true for N and P fertilisers). Another source of increasing pressure is the growing the crops for bioenergy. Again, it is the efficiency of the agricultural system that must be improved so as to avoid the transgression of this PB (Campbell et al., 2017).

#### 1.2.5 Land use

Land use change, specifically the clearing of forests for agricultural cropland and pastures, was assigned the safe limit of maintaining at least 75% of original forest land cover with a zone of uncertainty extending to maintaining just 54% of original forest cover. The current status of this PB control variable was determined to be 60% placing it within the zone of uncertainty with increasing risk (Richardson et al., 2023). A primary driver for land use change is increasing agricultural production to feed a growing global population. Estimates vary slightly, mainly due to the allocation of rangelands used intermittently for grazing, but it is largely accepted that around half of the Earth’s habitable area, or 40% of the total land area, is used for pastoral or arable farming (Ellis, Klein Goldewijk, Siebert, Lightman, Ramankutty, 2010, Engström, Gars, Krishnamurthy, Spiro, Calel, Lindahl, Narayanan, 2020, Ritchie, Roser, 2013).

**Fig. 5:**
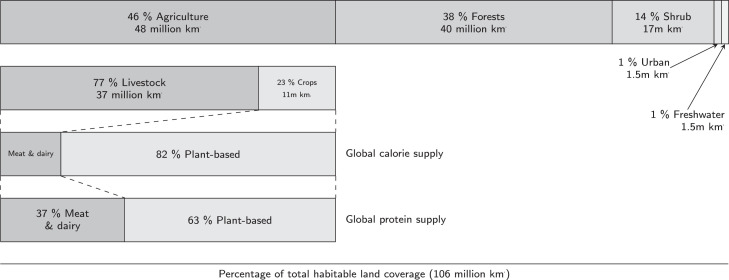
Land use as shown as proportions of the Earth’s total habitable land area of 106 million km${}^{2}$, not including barren land or glaciers which account for 28 million km${}^{2}$ (19%) and 15 million km${}^{2}$ (10%) of total land surface, respectively. Urban land includes settlements and infrastructure whilst freshwater coverage accounts for lakes and rivers. 77% of agricultural land is used for grazing lands and crops for animal feed. The remaining 23% of agricultural land is used to grow crops for food (not including feed). Despite covering 77% of agricultural lands, animal-sourced meat and dairy products provide only 18% and 3% of global calories and protein, respectively. Adapted from Ritchie and Roser (2013).

Fig. 5 shows the use of Earth’s habitable land area of 106 million km${}^{2}$. This area is found by subtracting the area covered by barren land (28 million km${}^{2}$) and glaciers (15 million km${}^{2}$) from the Earth’s total land surface (149 million km${}^{2}$) (Ritchie and Roser, 2013). 53% of the Earth’s habitable surface remains unaltered by anthropogenic activities, consisting of forests (38%), shrubland (14%), and lakes and rivers (1%). The remaining 47% of habitable land has been transformed from its natural state in order to support human communities, yet only 1% is covered by cities, towns, roads, industry, and other infrastructure. The data therefore implies that over 97% of anthropogenic land use is for agriculture. However, the explicit definition of the land use change PB as the conversion of forests results in agriculture’s contribution to the transgression of the PB of about 80% (Campbell et al., 2017).

Fig. 5 also highlights the proportion of agricultural land dedicated to livestock (including pastures and croplands for animal feed) and crops (solely for human food consumption). Specifically, of the 48 million km${}^{2}$ of agricultural land, 77% (37 million km${}^{2}$) is allocated to raising livestock whilst 23% (11 million km${}^{2}$) is for crops. The high land use intensity of meat and dairy products is demonstrated by the fact that despite accounting for 77% of agricultural land coverage, these food products only provide 18% of the world’s calories and 37% of total protein. The protein supply values suggest that, were a plant-based diet to be universally adopted as the only source of both calories and protein, the amount of land required for global food supply could be reduced by at least 64%, even without switching to higher protein crops.

#### 1.2.6 Summary of agricultural impacts

The environmental impacts of traditional agriculture, particularly animal-sourced food products, contribute significantly to the transgression of the PBs discussed. Agriculture contributes 25% of global GHG emissions, due to land use change (for raising livestock) and significant emissions of potent CH${}_{4}$ and N${}_{2}$O GHGs (from enteric fermentation and manure, respectively) (Poore and Nemecek, 2018). Agriculture has also been shown to contribute 85% and 90% of the N fixation and P loading PB impacts, respectively (as well as the associated environmental issues such as eutrophication and water toxicity), due to abundant and inefficient over-application of fertilisers which is exacerbated by crop-intensive livestock feeding regimes and waste runoff (Campbell et al., 2017). This inefficiency of growing crops for animal feed further drives the PBs of freshwater consumption (for irrigation) and land use change (for grazing and croplands) to which agriculture contributes 84% and 80%, respectively (Campbell et al., 2017).

Unfortunately, socioeconomic development is increasing global meat and dairy demand, and so exacerbating the associated environmental issues. An overhaul of the global food production system is necessary for an increasing global population to be sustainably fed. One solution would be adoption of circular economic principles where wasted resources are recovered and recycled to substitute a significant fraction of the primary production. Another potential solutions is adopting more efficient food production systems, particularly for protein production, such as insect cultivation, or microbial protein production via industrial fermentation, with a reduced land, water, nitrogen and phosphorus footprint compared to the current mix of farming methods.

### 1.3 Resource recovery & PB-LCA

The linear take-make-waste economy generates large amounts of waste and increases resource scarcity, as growing demand is met by increasing the consumption of raw materials and energy (Cong et al., 2017). Resource recovery enables a shift from the linear take-make-waste economy towards a more circular one that recovers value-added products from waste, enabling the increasing demands of economic growth to be met in an environmentally sustainable and resource efficient way (Lieder and Rashid, 2016). By returning recovered resources as feedstocks to the supply chain, demand for primary resource can be reduced. Recovering WEF resources from food and agricultural waste can mitigate the environmental impact of food production (Keijer et al., 2019).

There are clear links between the planetary boundary framework and decision-making in wastewater treatment and resource recovery (WWTRR). Decisions about WWTRR directly affect the biogeochemical cycle, freshwater extraction and climate change, as well as biosphere integrity and land-use. However, efforts to apply the planetary boundary framework to the WWTRR design problem are at an early stage. Ryberg et al. (2020) used a share of the safe operating space (SoSOS) approach to assess the absolute environmental sustainability assessment of a Danish water supply and wastewater treatment company, and Lima et al. (2023) used PB-LCA to assess the absolute sustainability of two alternative sanitation systems at a city-level. Both studies use an equal per capita approach to downscale PBs to a community-level, with a economic approach used to find the proportion of the total community PB to be attributed to WWTRR. However, to the best of the authors’ knowledge, planetary boundaries haven’t yet been used in WWTRR synthesis at a larger scale, or to systematically assess a wide range of WWTRR options.

Outside WWTRR, Algunaibet et al. (2019) demonstrated how the PB framework could be combined with LCA and SoSOS to address system design in the power sector, and Tulus et al. (2021) used a similar approach to assess the sustainability of chemical products in terms of PBs. Note that the latter work establishes a system to evaluate the environmental PB costs of producing a product, and this approach could be used to assess the benefits of resource recovery, which in PB-LCA are represented by larger SoSOS (Lima et al., 2023). A similar approach to Algunaibet et al. (2019) could be used to design an absolutely sustainable WWTRR system.

Wastewater is increasingly regarded as a source of valuable resources as opposed to an effluent necessitating costly contaminant removal (Villarín and Merel, 2020). Resource recovery from wastewaters has the potential to alleviate environmental problems and resource stress associated with the carbon, nutrient, and water cycles (Angenent et al., 2004). For example, water reclamation and the recovery of energy and valuable nutrients can alleviate water stress and dependence of fossil-based production, respectively. Additionally, recovery of nutrients from wastewater reduces emissions to the environment and corresponding issues such as eutrophication or aquatic toxicity. Within these wastewater streams are considerable carbon-containing and nutrient-rich resources which could be converted into biofuels (energy vectors) and value-added products, including food and feed, whilst simultaneously recovering the water for return to freshwater bodies. For example, oxidation of domestic wastewater organics has a maximum energy potential of 2.25 kWh m${}^{-3}$ (Wan et al., 2016), or 0.8 kWh${}_{e}$ (KWh${}_{e}$ - KWh of electricity) at 35% efficiency (Kabeyi and Olanrewaju, 2022), whilst the potential fossil energy displaced by recovering fertiliser elements (P/N) is 0.8 kWh m${}^{-3}$ (McCarty et al., 2011). Equally, co-producing fertiliser and biogas from biowaste produces fertiliser with a lower carbon footprint than conventional methods, at 0.81 kgCO${}_{2}$/kgN and 1.6 kgCO${}_{2}$/kgP, relative to 3.7 kgCO${}_{2}$/kgN and 3.1 kgCO${}_{2}$/kgP, savings of 3.1 kgCO${}_{2}$/kgN and 1.5 kgCO${}_{2}$/kgP (Havukainen et al., 2018). For typical domestic wastewater (COD = 500 g/m${}^{3}$, TN = 40 g/m${}^{3}$, TP = 8 g/m${}^{3}$ (McCarty et al., 2011), this means introducing biogas used for electricity production could deliver up to 375 g/m${}^{3}$ of carbon savings (based on average carbon intensity of electricity of 475 gCO${}_{2}$/kWh${}_{e}$ (IEA, 2019)), while nitrogen and phosphorus recovery account for up to 136 gCO/m${}^{3}$ of additional carbon savings. Furthermore, carbon emission from industry are often harder to abate than those from electricity production; industrial carbon emissions continue to rise, while emissions from electricity generation may have peaked (Ritchie et al., 2020a). Consequently, a paradigm shift is underway to shift the focus of wastewater treatment plants from end-of-pipe treatment towards sustainable resource recovery facilities (Guest et al., 2009).

Anaerobic digestion (AD), by far the most widespread recovery technology in the wastewater and bio-solid sector, converts sewage sludge organics into methane-rich biogas for energy production. China has over 100,000 anerobic digestion plants (Lu and Gao, 2021), producing approximately 72,000 TWh of biogas (Agency, 2023). However, there are concerns that the focus on energy could hinder the recovery of other, more environmentally valuable resources (HM Government, 2015). Technology innovation is underway to recover diverse value-added products from wastewaters, including thermochemical processes to convert AD digestate to biocrude (Eboibi et al., 2015) or reformation of AD-produced biogas to hydrogen for microbial protein production (Matassa et al., 2015). An integrated resource recovery process would produce a spectrum of bio-derived value-added products by incorporating multiple unit processes into a unified flowsheet (Cherubini, Jungmeier, Wellisch, Willke, Skiadas, Van Ree, de Jong, 2009, Rama Mohan, 2016). However, a major challenge is deciding which combination of processes are optimal in a given context (Guest et al., 2009).

Integrative resource recovery facilities can assist in alleviating stress on natural resources, by increasing the environmental sustainability of existing waste management practices and recovering bioproducts across the WEF nexus. These waste resource recovery facilities could also assist in catalysing a waste bioeconomy in which the inherent resource value is regenerated, thus contributing to the paradigm shift from a traditional linear economy towards a more circular one. By recognising the value embedded within waste streams, waste streams can be regarded as a source of economic value as opposed to an economic burden. In such a way, a bioeconomy in which the inherent value of bio-based wastes is quantified and traded could be realised. However, for this to be realised, the design of resource recovery systems, the characterisation of embedded resources available for recovery, and the recognition of the value of environmental goods needs to be addressed.

### 1.4 Food & drink manufacturing

Food and drink manufacturing is a large and economically important global industry. The global food and drink market is worth more than $9.1 trillion per year (Statista, 2024). In many regions, such as the US and the EU, food and drink manufacturing is the largest manufacturing sector by revenue (Bureau, 2021, Europe, 2023). However, major changes to both food and drink manufacturing, and the wider food and drink system will be needed in order to respect planetary boundaries. Food production currently accounts for over a quarter (26%) of global greenhouse gas emissions, occupies half of the world’s habitable land, consumes 70% of global freshwater withdrawals, and drives 78% of global ocean and freshwater eutrophication (Ritchie et al., 2022a). To address these environmental challenges, significant innovations in food and drink production, including within food and drink manufacturing, are essential.

#### 1.4.1 Fermentation & microbial food

One promising strategy to decrease the PB risks of the food system while simultaneously increasing food production is the use of alternative protein sources, such as microbial proteins. Microbial proteins provide a sustainable and nutritious alternative to traditional animal and plant-based proteins (Banks et al., 2022). For these environmental benefits to be impactful, however, microbial proteins must be widely adopted.

Specifically, microbial protein is produced in controlled environments, independent of animal husbandry. This enables technological process optimisation and significantly greater efficiency than animal agriculture, allowing for large-scale protein production without compromising environmental sustainability (Parodi, Leip, De Boer, Slegers, Ziegler, Temme, Herrero, Tuomisto, Valin, Van Middelaar, Van Loon, Van Zanten, 2018, Post, 2012). Benefiting from shorter production cycles, microbial protein offers a scalable solution providing protein security and resilience under extreme events, such as pandemics or droughts, which interrupt plant- and animal-sourced protein supply chains. Agricultural land can be made available by reducing the amount of crops required for human and livestock consumption (Matassa et al., 2023). More than 80 microbial strains including bacteria, microalgae and fungi/yeasts have been reported to enable the production of food-grade or feed-grade protein (Banks et al., 2022). Increasingly, genome sequencing and gene editing are being harnessed to select and engineer microbes for specific purposes (Pan and Barrangou, 2020). However, such methods raise concerns over safety, and come with significant economic costs during the research phase (Pan and Barrangou, 2020).

### 1.5 Summary of motivations

One of the greatest challenges facing humanity is meeting the growing demands of an increasingly developed global population whilst operating within the ecological limits of the planet. The PB framework quantifies these safe operating limits yet there remains the challenge of ensuring sustainable supply of water, energy, and food within these constraints. Traditional energy production (contributing over 90% of CO${}_{2}$ emissions) and food production (contributing 25%, 85%, 90%, 84%, and 80% to the PBs for climate change, N fixation, P loading, freshwater consumption, and land use change, respectively) have been highlighted as the primary drivers of PB transgressions. Therefore, simply ramping up food and energy production via traditional systems is unsustainable. In light of this, microbial food and resource recovery have been introduced as potential solutions to provide WEF resource security with reduced environmental impacts.

This work focuses on the recovery of treated water, energy, and nutrient resources from wastewater. Water is recoverable via wastewater treatment to meet effluent constraints for emission to receiving water bodies, reducing net consumptive use as measured by the freshwater PB. Energy resources are recoverable as solid, liquid, or gaseous energy vectors as well as via combined heat and power systems, with the energy recovered substituting a fraction of the demand for fossil fuels, addressing the climate change PB. Finally, N and P nutrients can be recovered from wastewater and recycled into the food production system, reducing its dependence on the highly-polluting, fossil-based production systems of these nutrients.

Wastewaters are inherently varied in their flows and composition. Food and beverage processing wastewaters (FPWW) are the focus of this work due to the connection with WEF resources. Furthermore, FPWW could be seen to represent the low-hanging fruit of wastewater resource recovery, as they are often high in concentrations of potentially recoverable carbohydrates, proteins and fats, and may be lower “entrop” than municipal wastewater. Additionally, where WWTRR plants are co-located and co-operated with food and beverage processing facilities, the processes could be co-optimised, as some of the incentive problems of municipal WWTRR plants are avoided. Of particular interest are wastewaters from fermentation-based food and beverage production. The controlled operating environments for fermentation-based food and beverage production enables optimisation of operating parameters. Producing food in this way, coupled with resource recovery from FPWW is a potential solution for the sustainable provision of WEF resources in relation to the PBs.

Realising these systems, with WEF resources recovered from fermentation-based FPWW, requires a holistic, systems-orientated process design methodology to balance multiple competing decision criteria. Process systems engineering (PSE) provides a scientific discipline and computational methodologies, particularly with recent advances in artificial intelligence and machine learning, to address this challenge (Pistikopoulos et al., 2021).

## 2 Literature review - from wastewater treatment to resource recovery

“Un égout est un malentendu.” “A sewer is a misunderstanding” *Les Misérables*, Victor Hugo.

### 2.1 From carbon source to carbon sink

In order to avoid breaching PBs, we need to shift from seeing wastewater as a waste to be treated to seeing it as a resource to be processed into useful products. This paradigm shift should also see WWTRR transition from being a carbon source to a carbon sink. While the IPCC recommends that the carbon dioxide emissions from oxidation of organic carbon in wastewater should generally not count as additional, as the carbon is usually biogenic, coming from food waste or excrement (Li et al., 2022), conventional treatment demands electrical energy (0.4-1 kWh/m${}^{3}$ (Ranieri, Giuliano, Ranieri, 2021, Wan, Gu, Zhao, Liu, 2016)), and generating this energy usually results in additional carbon emissions (global average: 475 gCO${}_{2}$/kWh (IEA, 2019)).

However, recovering resources, such as biogas, nitrogen and phosphorus, from wastewater could allow WWTRR to become carbon-neutral or even carbon-negative. The extractable energy present is wastewater has been estimated at 2.25 kWh/m${}^{3}$ (Wan et al., 2016), which is more than sufficient to cover the energy required for operation, even allowing for conversion losses associated with generating electricity.

Under the status quo, much of the organic carbon present in sewage and sewage sludge is emitted to the atmosphere as carbon dioxide or methane, without energy extraction. The emissions of land application and landfill of sewage sludge have been estimated at 2–3 tons of CO2 -eq per ton of dry sludge, and 7 tons of CO2 -eq per ton of dry sludge respectively (Pilli, Bhunia, Yan, Tyagi, Surampalli, 2014, Zhou, Yang, Zhao, Dong, Wang, Wei, Yang, Sun, Ren, Bai, 2023). Compared to this, generation of bio-fuels, such as bio-gas or bio-oils, from sewage is an attractive option, as these can be used as a substitute for fossil fuels, reducing net carbon emissions. Alternatively, sewage sludge can be used to produce biochar for carbon sequestration in the soil (Lu et al., 2018). This improves the proportion of the carbon that is retained in the soil compared to direct landfill or land application, which are still the most common end-destinations for sewage sludge in both Europe and the US (Kelessidis, Stasinakis, 2012, Krause, Bronstein, 2024).

### 2.2 Wastewater characterisation

As wastewater treatment undergoes a paradigm shift from contaminant removal towards sustainable resource recovery, the characterisation of different wastewaters needs to become more detailed to determine the potential for resource recovery. Traditionally, wastewater characterisation has focused on characterising the amount of organic matter present using chemical oxygen demand (COD) and biochemical oxygen demand (BOD), which are measures of the amount of oxygen required to remove the organic compounds; this definition alone shows the traditional way of thinking about organics as something to be destroyed by aerobic method). The total suspended solids (TSS), total phosphorus (TP), and total nitrogen (TN) content were also measured to ensure the treated effluent met environmental constraints prior to release to receiving water bodies. The removal of nitrogenous and phosphoric compounds was first required due to their potential to cause eutrophication (in the case of N and P) and aquatic toxicity (in the case of ammonia) (Puchongkawarin et al., 2015). Because of these traditional characterisations, the most recognised products available for recovery are water, biogas from anaerobic digestion (AD) of organics, and organic fertilisers rich in N and P (Verstraete et al., 2009). Table 2 shows typical ranges for municipal and select industrial wastewater concentrations.

**Table 2:** Wastewater characterisation by source.

Wastewater	COD	BOD	TSS	TP	TN	pH	Ref
	(mg L${}^{-1}$)	(mg L${}^{-1}$)	(mg L${}^{-1}$)	(mg L${}^{-1}$)	(mg L${}^{-1}$)		
Municipal	450–800	350–600	200–450	4–15	35–60	6.0– 7.8	(Von Sperling, 2007)
Textile	1 500–3 100	1 850	150–300	0–3	17–23	9.0–13.0	(Sahinkaya et al., 2008)
Brewery	2 000 – 6 000	1 200–3 600	2 900–3 000	10–50	25–80${}^{*}$	3.0–12.0	Simate et al. (2011)
Paper Mill${}^{+}$	3 380–4 300	1 650–2 570	1 900–3 140	N/A	N/A	6.2– 7.8	Zwain et al. (2013)
Pharma${}^{\#}$	4 650–8 850	2 150-2 350	1 200	N/A	380–440${}^{*}$	3.9– 9.2	Gadipelly et al. (2014)
Dairy	5 000–8 000	3 000–5 000	N/A	50–70	50–150${}^{*}$	4.0– 7.0	Sirianuntapiboon et al. (2005)

Where two values are given, these represent the typical range of values for the type of wastewater, based on the corresponding reference. Single values are typical values. N/A - Not available. *Total Kjeldahl Nitrogen, #Fermentation & chemical processes, + Recycled Paper Mill.

As the wastewater treatment sector shifts to a resource recovery focus, the development of more detailed characterisation processes has begun Choubert et al. (2013), Tran et al. (2015), Ravndal et al. (2018). Specifically, more detailed characterisation methods of new variables, fractions, resource recovery processes, and operating conditions have been highlighted as a requirement for applying more granular modelling techniques to wastewater systems. Furthermore, more frequent sampling and standardisation of methods may be needed to facilitate data analytics and modelling (Choubert et al., 2013). In addition, deeper understanding of existing variables in the context of resource recovery can be used to optimise facilities (Tran et al., 2015). Finally, combinations of emerging methods can also be used, such as combined size fractionation, chemical composition and biodegradability analyses to categorise organic matter into carbohydrates, proteins, lipids, oils and fats, and fatty acids (Ravndal et al., 2018). Such granular characterisation enables additional product availability to be assessed during the design of resource recovery facilities, namely: protein for animal feed, lipids for biodiesel production, and removal of oils and fats which may damage downstream process units. By correlating particle size distributions with composition and biodegradability analyses, specific size fractions can be targeted for optimal separation and recovery.

In addition to chemical composition analyses for assessing physico-chemical and biological treatability, proximate and ultimate analyses are relevant for thermochemical processing. Proximate analysis shows the volatile matter and fixed carbon fractions whilst ultimate analysis detailed the elemental compositions, e.g., carbon (C), hydrogen (H), oxygen (O), nitrogen (N), sulphur (S), chlorine (Cl) (Sadhukhan et al., 2014). The elemental analyses can provide insights into recovered biofuel quality as lower O/C and H/C ratios increase the energy value of fuel closer to that of commercial coal (McKendry, 2002). Additionally, elemental characterisation enables crude approximations of more value-added products such as the estimation of protein by multiplying the N composition by a conversion factor (Mariotti et al., 2008).

Unlike municipal wastewaters, which can be generalised using a typical wastewater characterisation, industrial wastewaters are highly dependent on the industry as well as local site operations, resulting in much higher variations in compositions (Table 2). Additionally, the dependence on the upstream industrial practices results in flows, compositions, temperature, and pH exhibiting large temporal, as well as spatial, variations as many processing sites operate on a shift basis (Ng, 2006).

Due to the large range in industrial wastewater composition, it is necessary to focus on a particular industry for the purposes of this review. The UK food and beverage industry is the largest manufacturing sector in the UK (20% of total manufacturing turnover) and is projected to grow rapidly in order to feed a growing population of 70 million by 2024 (Food and Drink Federation, 2020). However, rapid growth has been accompanied by rising waste and resource use (WRAP, 2020) as well as demand for animal products and processed food and beverages leading to severe environmental concerns (United Nations Environment Programme, 2021). The industry is also a major water consumer (190 million m${}^{3}$ y${}^{-1}$ in the UK (Ajiero and Campbell, 2018)), generating a significant quantity of FPWW of varying composition. Despite the increasing research attention on resource recovery from organic solid waste (Kumar and Samadder, 2017), including solid by-products from the food industry e.g., Quorn mycoprotein waste used directly as animal feed (Finnigan et al., 2010) or brewer’s spent grain fermented to single cell protein (SCP) or processed as additives (Bianco et al., 2020), efficient valorisation of liquid FPWW remains an open challenge.

There are considerable benefits in recovering energy and protein from various FPWWs (Asgharnejad, Khorshidi Nazloo, Madani Larijani, Hajinajaf, Rashidi, 2021, Durkin, Guo, Wuertz, Stuckey, 2022, Grünwald, 2021). Table 3 summarises the resource recovery potential from different FPWW, where the COD and TN contents were used to approximate theoretical maximum electrical energy recovery and protein recovery, respectively. The potential 425 TWh recoverable from FPWW could theoretically supply more than 1% of global electricity demand (Ritchie et al., 2020b), or more than half of the global electricity supplied by photovoltaic installations in 2020 – 821 TWh (IEA, 2021). Similarly, the potential 21.4 Mt of annual protein equivalent could theoretically supply all of the animal feed consumed by global aquaculture - approximately 20.6 Mt y${}^{-1}$ (International Feed Industry Federation, 2021). For reference, global food-grade protein demand is about 203 Mt Protein y${}^{-1}$ (Henchion et al., 2017), while demand for high-protein feed is about 1170 Mt y${}^{-1}$ (International Feed Industry Federation, 2021), with typical protein concentrations of 50–60% (Schlageter-Tello et al., 2020), suggesting feed-grade protein demand of about 640 Mt Protein y${}^{-1}$. Recovering energy and protein from FPWW could be facilitated by reducing the volume of water used in processing food, to increase FPWW nutrient concentrations, which would have the additional benefit of reducing water consumption (Asgharnejad et al., 2021).

**Table 3:** Resource recovery potential from different food processing wastewaters. Chemical oxygen demand was used to estimate theoretical potential for electrical energy recovery, whilst total nitrogen was used to estimate theoretical potential for protein recovery.

Food Processing Industry	Global Wastewater Production	Chemical Oxygen Demand	Potential Electrical Energy Recovery		Total Nitrogen	Potential Protein Recovery	
	(million m${}^{3}$ y${}^{-1}$)	(g m${}^{-3}$)	(GWh y${}^{-1}$)	(% of Global Demand)	(g m${}^{-3}$)	(kt y${}^{-1}$)	(% of Global Demand)
Fish, Meat & Dairy	2 650	2 680	11 200	0.04	243	4 020	1.98
Fruit & Vegetables	6 435	18 100	183 000	0.62	236	9 490	4.67
Edible oils	1 470	29 600	68 500	0.23	790	7 260	3.58
Grain milling	3 380	27 300	145 000	0.49	N/A	N/A	N/A
Bakery	457	6 510	4 690	0.02	56	159	0.08
Sugar, Tea & Coffee	485	7 145	5 460	0.02	24	73	0.04
Brewing	1 210	4 000	7 620	0.03	53	400	0.20
Aggregate	16 100	16 800	425 000	1.44	213	21 400	10.5

Except for potential electricity generation, and brewing figures, all values are based on Durkin et al. (2022b). Brewing figures are based on Simate et al. (2011) and Barth-Haas Group (2022). Potential electrical energy has been calculated on the basis of 16.2 kJ/gCOD (Wan et al., 2016), and electric conversion efficiency of 35% (Kabeyi and Olanrewaju, 2022) for internal consistency. Protein potential is based on 6.25 g protein / g TN (Mariotti et al., 2008). Some industries have been aggregated together, such as Fish, Meat & Dairy, which were treated as 6 different industries in Durkin et al. (2022b). Where this has been done, the COD & TN values given are averages of the values of the constituents weighted by the volume of wastewater produced. Weighted averages are also used for TN and COD in the aggregate row. Wastewater production, potential energy & protein recovery, are simple sums. Protein as a proportion of global demand is based on annual global demand of 203 MT (Henchion et al., 2017). Electrical energy recovery as a proportion of total global demand is based on annual global demand of 29,479,000 GWh (Ritchie et al., 2020b).

Despite its potential, resource recovery from FPWW is rarely implemented, with the wastewater instead often disposed of to sewers, or occasionally converted to low value energy via AD and combined heat and power (CHP) systems to low value methane, ethanol or hydrogen (Batstone, Virdis, 2014, Uckun Kiran, Trzcinski, Ng, Liu, 2014). One of the key challenges is that the specific chemical composition of complex FPWWs remains largely undefined (Environment Agency, 2015), and their full potential unknown because analysts are unused to carrying out “untargeted analysis” where they do not know exactly what to look for. Untargeted analysis is time consuming and sometimes difficult, but as more is done it is possible to catalogue the compounds identified and understand their economic use further enabling the advancement of our understanding of the resource recovery potential at a global scale. However, the crudeness of current FPWW characterisation limits understanding of resource recovery potential.

### 2.3 Conventional wastewater treatment

Traditional wastewater treatment plants focus on removing organic carbon as well as nitrogenous and phosphorus compounds from wastewaters so as to meet environmental constraints on the quality of the final effluent emitted to a receiving water body. The most established technologies exist within a treatment line categorised into 5 stages: preliminary treatment, primary treatment, secondary treatment, tertiary treatment, and sludge treatment. Preliminary treatment is designed to remove large or inert matter, such as rags or grit, from the influent. Oil and grease are also removed here by oil and grease traps, so that they do not damage downstream processes or interfere with settling processes (Ng, 2006). Additionally, equalisation tanks enable temperature and pH control as well as mixing to produce compositions closer to average values used in the plant design (Ng, 2006). These also serve as buffer tanks for the downstream processes to be operated continuously despite upstream industrial processes operating in shifts.

Primary treatment removes the settleable solids with clarifiers or fine screens, producing a thickened primary sludge stream alongside the main clarified effluent that is fed to the next stage of the process. Secondary treatment is typically an aerobic process followed by secondary clarifiers, where the aim is to remove the organic matter via biological degradation. Finally, if required, tertiary treatment is employed to disinfect and remove any remaining contaminants prior to being emitted to the receiving water body. The main problems associated with this traditional treatment pathway are: the generation of large amounts of sludge which requires further processing; large energy requirements for the aeration of secondary aerobic treatment processes (McCarty et al., 2011); and failure to recover potential resources available in the wastewater (Stazi and Tomei, 2018).

One of the most widely adopted treatment processes is the conventional activated sludge (CAS) process due to its mature status developed over 100 years. However, secondary aeration and settling processes within CAS configurations are responsible for high capital costs and large land footprints, high operational costs due to high aeration requirements, and high sludge production, all whilst failing to recover any value-added resources (Nancharaiah et al., 2019). Specifically, CAS processes are configured as a secondary treatment process with a biological aeration tank followed by secondary clarification. Secondary clarification produces a treated effluent overflow and two activated sludge streams: waste activated sludge (WAS) is typically combined with primary sludge and fed to AD-CHP systems to recover low-value energy; whilst return activated sludge (RAS) is recycled to biological treatment in the aeration tank.

Such wastewater treatment plants were designed with 3 objectives: to remove contaminants, ease effluent and sludge management, and meet legislative effluent quality constraints (Raheem et al., 2018). As such, high capital and operating costs were accepted to achieve efficient contaminant removal with no regard for resource recovery opportunities other than low-value AD-CHP. The sustainable revolution of wastewater treatment towards resource recovery has shifted the goals of plant design: towards sustainable resource recovery of value-added products and the production of environmental goods.

Despite the shortcomings of CAS, a group of mathematical models, under the generic name activated sludge model (ASM), have been developed due to the prevalence of CAS in industry and the importance of simulating and optimising operations (Henze et al., 2006).

### 2.4 Anaerobic digestion

**Fig. 6:**
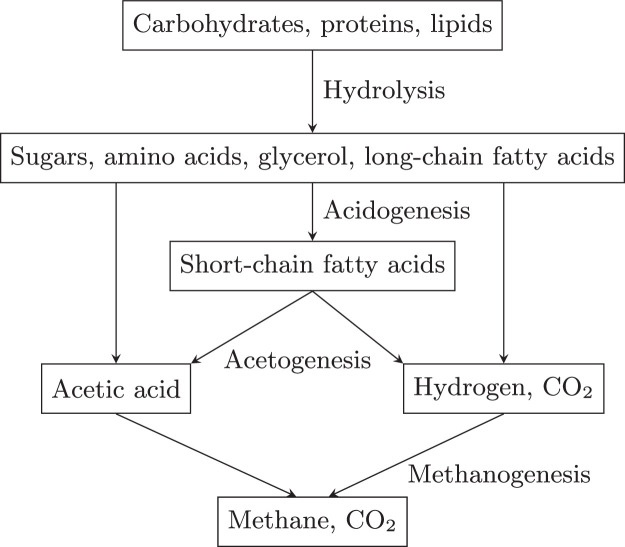
Four stages of AD mechanism: hydrolysis, acidogenesis, acetogenesis, and methanogenesis.

Anaerobic Digestion (AD) has received much development over previous decades as a method to recover biogas, and ultimately electricity via CHP processes, from wastewater organics. AD is a process in which bacteria break down organic matter, in the absence of oxygen, to produce biogas rich in methane and carbon dioxide. A byproduct of AD is the decomposed substrate, referred to as a digestate, which is rich in organic matter, nitrogen, and phosphorus, making it useful as a fertiliser. However, concerns have been raised over the potential for digestate to contain pathogens, heavy metals, and other potentially toxic elements. The AD mechanism can be classified into four sequential processes in which each process uses the products of the former process as substrates. These processes are hydrolysis, acidogenesis, acetogenesis, and methanogenesis which proceed as shown in Fig. 6.

The hydrolysis stage breaks down large organic polymers such as carbohydrates, proteins and fats, into smaller monomers such as sugars, amino acids, glycerol, and long-chain fatty acids. Following this slow hydrolysis step, acidogenesis converts the smaller organics into volatile fatty acids (VFAs) such as propanoic acid and butyric acid. These acids are then used by acetogenic bacteria to produce acetate, hydrogen, and carbon dioxide. Finally, methane is produced by methanogenic bacteria breaking down acetic acid, in the absence of oxygen.

AD is a sensitive process due to the difficulty balancing the rates of different bacterial reactions (hydrolysis, acidogenesis, acetogenesis and methanogenesis) to maintain steady concentrations of intermediates, which is complicated by the inherent fluctuations in wastewater compositions and flows. Important parameters that must be controlled include temperature, pH, hydraulic retention rate (HRT) and solids retention time (SRT). HRT refers to the amount of time that the bulk liquid spends in the reactor, whilst SRT refers to the amount of time solids are retained in the reactor. Sudden changes in HRT and SRT can result in accumulation of VFAs due to decreased methanogenesis compared to acidogenesis. This leads to a decrease in pH and alkalinity, and ultimately results in a biogas with reduced a methane composition in favour of carbon dioxide and hydrogen. The primary inhibitory compounds for the methanogens in AD are ammonia (Yenigün and Demirel, 2013), xenobiotics, heavy metals, detergents, and of course oxygen (Leitão et al., 2006).

Anaerobic sludge digestion is widely used in the EU and is the favoured method in Spain, UK, Italy, Finland and Slovakia (Kelessidis and Stasinakis, 2012). The methane gas is converted by CHP plants to produce electricity and heat. This energy production can offset some of the CAS process energy demands whilst also providing a sludge remediation pathway, thereby addressing some of the problems with traditional wastewater treatment. However, if AD is only used to treat sludge, it does not realise the energy potential contained in the wastewaters dissolved organic fraction, which is still treated via expensive aerobic processes resulting in net energy consumption (McCarty et al., 2011).

More energy could be recovered using complete anaerobic digestion of wastewater, which would increase organic matter to methane conversion and eradicate the intensive aeration energy requirements, resulting in net energy production when coupled with CHP (McCarty et al., 2011). Anaerobic digestion also has little impact on the ammonium and phosphate concentrations in wastewater, enabling nutrient resource recovery processes to be employed downstream (Puchongkawarin et al., 2015).

However, conventional AD processes also have their downsides, such as sensitivity to external parameters and long residence times, leading to large plant footprints. Some of these drawbacks, particularly the requirement for long residence times, have been addressed by architectural innovations in AD reactor design, resulting in high-rate anaerobic reactors. High-rate anaerobic reactors can accommodate high volumetric loading rates and deliver high organic matter degradation rates by decoupling biomass SRT and wastewater HRT (Barber and Stuckey, 1999). It should be noted however, that these anaerobic systems typically do not remove the organic content as effectively as their aerobic counterparts and so post-treatment is often required to meet effluent constraints (Chernicharo et al., 2015).

The main reactor configurations for high-rate anaerobic reactors, along with the advantages and disadvantages of each, are given in Table 4. Overall, the choice of high-rate anaerobic reactor configuration depends on the specific requirements of the application, such as the type and strength of the wastewater, the desired effluent quality, and the available space and resources. A careful evaluation of the strengths and weaknesses of each configuration is essential for selecting the optimal system for a given application. Additionally, operational and maintenance considerations should also be taken into account when designing and implementing high-rate anaerobic treatment systems.

**Table 4:** Advantages & Disadvantages of Different Anaerobic Reactor Types.

Summary of Advantages & Disadvantages of Different Anaerobic Reactor Types Reactor				
Reactor	Description	Advantages	Disadvantages	Ref
Anaerobic Filter (AF)	Vertical filter beds of inert media provide support for biomass and entrap unattached flocs of organisms. Influent is pumped up through support media, allowing contact between microorganisms and wastewater.	Simple construction, Stable performance at high loading rates, Can withstand large toxic shock loads.	Biomass accumulation in support media can led to clogging. Large reactor volumes required to accommodate packing. Poor effluent quality relative to conventional aerobic treatment.	(Kobayashi, Stenstrom, Mah, 1983, Switzenbaum, 1983)
Anaerobic Fluidised Bed Reactor (AFBR)	Wastewater flows up through a column, fluidising a bed of inert sand-sized articles that provide support for microorganisms. Effluent recycling is used to maintain high upflow velocities for fluidisation.	Higher biomass retention capacity provides enhanced efficiency and stability. High organic loading rate can be accommodated thanks to large specific surface area of media.	Energy-intensive re-circulation of effluent may be necessary to maintain bed expansion. Poor effluent quality relative to conventional aerobic treatment.	(Heijnen, Mulder, Enger, Hoeks, 1989, Rajeshwari, Balakrishnan, Kansal, Lata, Kishore, 2000, Switzenbaum, 1983)
Upflow Anaerobic Sludge Blanket (UASB)	Wastewater flows up through a dense blanket of flocculated sludge at the bottom of the reactor. A gas-solid-liquid separator at the top of the reactor separates the biogas and ensures sludge retention.	Good exploitation of reactor volume, as no packing material needed. Good performance at high organic loading rates. Low susceptibility to wash-out once established.	Susceptible to temperature and organic shock loads. Washout, flotation and disintegration of granular sludges are possible. Dead spots in sludge blanket can reduce efficiency. Long start-up period. Requires significant amount of seed sludge for fast start-up. Poor effluent quality relative to conventional aerobic treatment.	(Chernicharo, van Lier, Noyola, Bressani Ribeiro, 2015, Chong, Sen, Kayaalp, Ang, 2012, Stazi, Tomei, 2018, Switzenbaum, 1983)
Expanded Granular Sludge Bed (EGSB)	Similar to UASB, but with taller reactors and an expanded sludge bed to increase waste-water-biomass contact and intensify hydraulic mixing.	Improved mixing relative to UASB results in better sludge-wastewater contact.	Start-up susceptible to temperature and organic loading shock. Maintaining upflow velocities requires energy-intensive effluent re-circulation. Washout, flotation and disintegration of granular sludge remain possible risks. Poor effluent quality relative to conventional aerobic treatment.	(Kato, Field, Versteeg, Lettinga, 1994, Seghezzo, Zeeman, Van Lier, Hamelers, Lettinga, 1998)
Anaerobic Baffled Reactor (ABR)	Composed of series of baffles that cause biomass to continually rise and settle due to flow characteristics and gas production.	Solids are retained in reactor for longer, independent of bulk wastewater flow. Offers good resilience to hydraulic & organic shocks, high biomass retention and ability to separate the phases of anaerobic catabolism.	Reactors must be shallow to maintain acceptable upflow velocities. Issues with maintaining even distribution of influent. Poor effluent quality relative to conventional aerobic treatment.	(Barber and Stuckey, 1999)
Anaerobic Membrane Bioreactor (AnMBR)	Uses external membrane module under pressure downstream of bioreactor to improve effluent quality.	Total biomass retention, improved effluent quality, small footprint, low sludge production.	Susceptible to membrane fouling by precipitation of struvite and calcium carbonate. Poor effluent quality relative to conventional aerobic treatment.	(Liao, Kraemer, Bagley, 2006, Ozgun, Dereli, Ersahin, Kinaci, Spanjers, Van Lier, 2013)
Submerged Anaerobic Membrane Bioreactor (SAnMBR)	Similar to AnMBR but operates under vacuum with membrane placed inside bioreactor liquid.	Total biomass retention, improved effluent quality, small footprint, low sludge production.	Immersed membrane is difficult to clean, exacerbating fouling issues. High costs. Poor effluent quality relative to conventional aerobic treatment.	(Hu, Stuckey, 2006, Ozgun, Dereli, Ersahin, Kinaci, Spanjers, Van Lier, 2013)

### 2.5 Resource recovery technologies

**Fig. 7:**
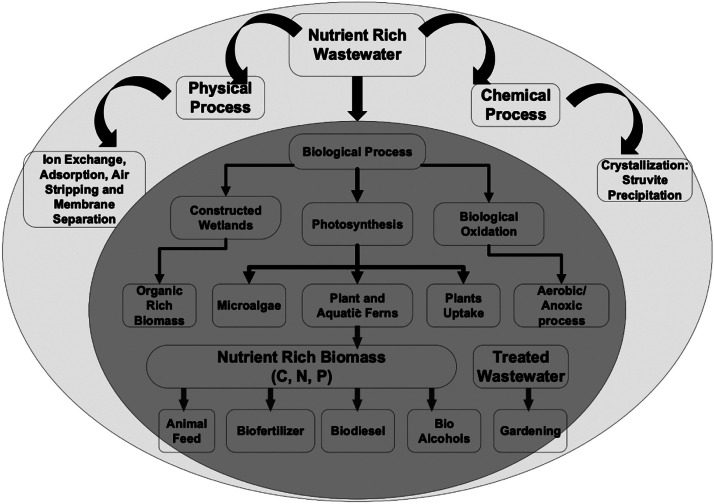
Technologies for nutrient recovery, focusing on biological methods. Diagram from Kong (2018).

There has been widespread technology development to recover different resources from wastewater via physico-chemical, biological, and thermochemical processing routes. Fig. 7 gives an overview of technologies for nutrient recovery, focusing on biological methods. Such technologies can be used to recover higher value products, compared to electricity and thermal energy recovered from traditional AD-CHP systems, such as different solid, liquid, and gaseous fuels, value-added chemicals, feed for animal consumption, and even food for human consumption.

**Fig. 8:**
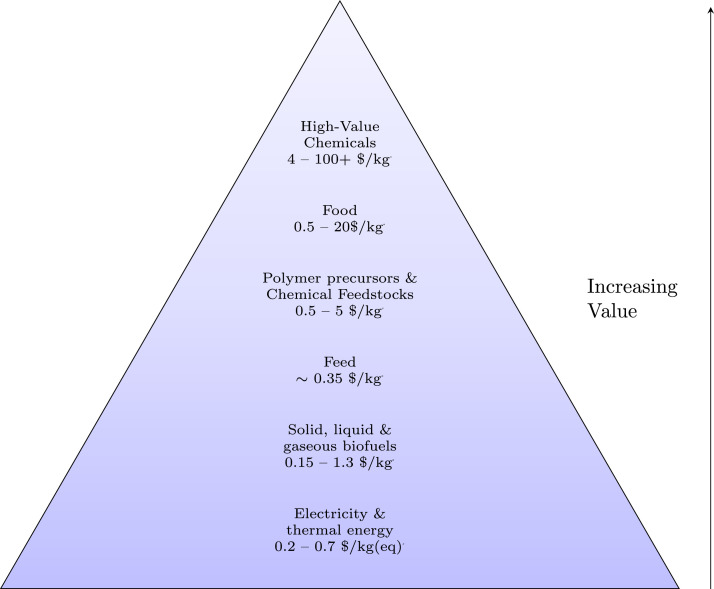
Biomass value pyramid showing approximate values of different bio-derived or bio-derivable products. Higher-value products appear further up the pyramid. Generally, the volumes consumed in mass terms are lower for higher-value products. Values are based on international commodity prices, except where otherwise indicated. A - Paracetamol 4 $/kg (Hill et al., 2018), Vitamins 13 $/kg (IndexBox, 2023), Penicillin 76 $/kg (Hill et al., 2018), Protease 9.50 $kg (Procurement Resource, 2023), Quinine 116 $/kg (Hill et al., 2018) B - Rice: 0.5 $/kg (Index Mundi, 2023), Myco-protein $22/kg (Retail Price) (Upcraft et al., 2021) C - Lactic Acid 0.97 $/kg (Research, 2023), Terephthalic Acid 0.65 $/kg (AgileIntel Research (ChemIntel360), 2023b), (AgileIntel Research (ChemIntel360), 2023a), Aldaric Acids 2–5 $/kg (Werpy and Petersen, 2004) D - Average Feed Price, calculated as Global Feed Market Value ($)/ Global Feed Production (kg) (International Feed Industry Federation, 2021) E - Natural Gas (US) 0.2 $/kg (World Bank, 2023), based on density of 0.657 kg/m3, Bio-diesel 1.3 $/kg (Comision Nacional de los Mercados y la Competencia, 2023) based on density of 0.85 kg/l, Coal 0.15 $/kg (World Bank, 2022) F - Thermal Energy - Natural Gas (US) 0.2 $/kg (World Bank, 2023), Electrical Energy 0.7 $/kg based on US industrial price of 0.085 $/kWhe (Administration, 2023) and electrical generation by combustion of natural gas at 50% efficiency (UK Department for Business and Strategy, 2022).

Fig. 8 shows different bioproducts recoverable or synthesisable from the organic fraction of wastewaters. The wastewater is first processed into intermediate platforms (sludge, biogas, syngas, biocrude) that are then refined to higher value products. This is analogous to the traditional oil refinery in which the crude oil is first fractionated into constituent hydrocarbon chain lengths ready for further reformation to different purpose fuels or chemicals. Increasing bioproduct value is depicted by a higher position in the value pyramid: for example, electricity and heat production, such as through use of a CHP unit, presents a low added-value solution, but with relatively low research and capital expenditure costs; production of high-value chemicals, food, feed or chemical feedstocks present solutions with a greater value-add, but would likely require more research and capital expenditure to establish at scale.

The design of waste biorefineries, incorporating multiple technologies to produce a spectrum of bioproducts, requires the following bottlenecks and trade-offs to be addressed: which recoverable products to prioritise; which technology to incorporate to extract specific products; in what configuration the technologies should be placed within an integrative flowsheet; and at what stage to stop reforming intermediate platforms. These competing technologies and bioproducts also have to be considered in conjunction with water reclamation; therefore, tertiary treatment becomes another decision variable, depending on whether further disinfection is necessary to meet effluent constraints.

#### 2.5.1 Physico-chemical technologies

For the production of most value added organic compounds, separation and purification is often a large fraction of the total cost, and because of their structure, most of these separation techniques come from biotechnology. These include membrane technology, foam fractionation, precipitation, adsorption, solvent extraction, and aqueous two-phase systems, and are an important part of adding value to FPWW (Lee and Stuckey, 2022). Separation can occur in two ways; from the original FPWW, e.g., protein (Osman, Gringer, Svendsen, Yuan, Hosseini, Baron, Undeland, 2015, Yadav, Yan, Ajila, Bezawada, Tyagi, Surampalli, 2016), or by fermenting FPWW to SCP and high added-value products such as Vitamin K2 (Ren, Peng, Hu, Han, Huang, 2020, Yadav, Yan, Ajila, Bezawada, Tyagi, Surampalli, 2016). Since most of the products from this stage will either be animal feed products, or dietary supplements, the purity required will not have to meet standards imposed in the U.S. by the Food and Drug Administration (FDA) and hence can be around 95%, provided toxic impurities are avoided.

Specific separations include using membranes to separate proteins from whey (Osman, Gringer, Svendsen, Yuan, Hosseini, Baron, Undeland, 2015, Yadav, Yan, Ajila, Bezawada, Tyagi, Surampalli, 2016), potato protein from FPWW (Dabestani et al., 2017), foam fractionation of isoflavone aglycones (Liu et al., 2013), size exclusion of nicotinamide mononucleotide (NMN) from bacterial cells (Marinescu et al., 2018), solvent extraction for citrus flavonoids (Sharma et al., 2019) and vitamin K2 (Berenjian et al., 2014), reactive extraction of gallic acid (Rewatkar et al., 2017), surfactant precipitation of proteins (Wong et al., 2018), and recovery of lactose and proteins using aqueous two-phase extraction (González-Amado et al., 2021). However, more work is needed to develop separation flowsheets for specific food solutes, and tailor separation techniques to meet the specific challenges of FPWW.

Physico-chemical separation of nitrogen/phosphorus can also be achieved using zeolite adsorption (Wang and Peng, 2010) or reactive sand filtration (Blaney, Cinar, SenGupta, 2007, Newcombe, Rule, Hart, Möller, 2008) respectively. Additionally, ammonia can be removed using air stripping methods followed by absorption into acid (Matassa et al., 2015). These methods separate the nutrients into a more recoverable form, compared to traditional biological enrichment of sludge, enabling higher-value product recovery with greater potential uses. For example, adsorbed N and P can be easily desorbed using concentrated brine solution allowing for production of fertilisers or SCP (using nitrogen source) for animal feed (Matassa et al., 2015).

Chemical precipitation and subsequent recovery reactions can be used to recover proteins, ammonium nitrogen and phosphorus from wastewaters. Recovered proteins can be used as high-grade animal feed whilst phosphorus is recovered in the form of metal phosphates. Nitrogen in the form of ammonium, and phosphorus, can be precipitated as struvite or magnesium ammonium phosphate (MAP), premium slow-releasing fertilisers.

Phosphates can be recovered from wastewater via chemical precipitation into metal phosphate salts and flocculation. Dosing can occur prior to primary sedimentation, during aeration processes, or post-secondary clarification. Additionally, dosing amount depends on the amount of dissolved phosphorus present in the wastewater. Based on stoichiometry, a 1:1 molar ratio of alum or ferric can remove 1 moe of phosphorus. In practice, excess dosing loads are typically used to achieve satisfactory precipitation.

Precipitation of struvite and MAP actually occurs naturally in wastewater treatment plants where it is considered a hindrance due to scaling and clogging issues. However, the industrial application of struvite/MAP precipitation processes is hampered by several reaction parameters requiring optimisation for specific FPWW streams, namely: pH control to manage alkalinity; magnesium dosage and magnesium-nitrogen ratio; initial ammonium nitrogen concentration; and different interfering ions (Zhang et al., 2011). The effectiveness of the recovered fertilisers is also strongly affected by the recovery process conditions such as pH, temperature, duration and additives used (Wollmann et al., 2018).

Phosphates can be recovered via ion exchange processes using ferric and aluminium salts (e.g., iron chloride, aluminium sulphate) as well as lime or poly aluminium chloride (PAC) (Li, Liu, Liu, Liu, Feng, 2017, Wang, Song, Lu, Zhao, 2014). Chemical phosphate removal is a complex process involving various steps, for example, when ferric chloride is used, the following mechanisms are involved: precipitation of ferric hydroxide, ferric phosphate and ferric-oxo-hydroxo-phosphate complex; phosphate adsorption on ferric hydroxide; and coagulation, flocculation and settling phenomena (Caravelli et al., 2012). Important control parameters in these processes are Fe:P ratio, pH, activated sludge biomass concentration, settling time and sludge age (Caravelli et al., 2012). Following phosphate precipitate recovery, P and pyrite (FeS${}_{2}$) can be recovered using acid and sulphide (H${}_{2}$S) dosing and precipitation (Mejia Likosova et al., 2013).

Once all the added value has been extracted from the FPWW in the form of valuable solutes and SCP, the residue of very mixed FPWW (high entropy FPWW) can then be treated so the water can be recycled. An energy efficient flowsheet could include an anaerobic membrane bioreactor (Shahid et al., 2020) as it maximises energy recovery (via biogas), and minimises the effluent COD and solids. With further post-treatment, such as adsorption and or ion exchange, the effluent could be recycled back into the process for water reuse, or discharged (Mai et al., 2018).

#### 2.5.2 Biological technologies

Biological reactors can also be used to recover nutrients (P/N rich components) via biological nutrient removal (BNR) and energy (in the case of AD). These biological processes can be categorised as aerobic, anaerobic or anoxic, where the latter is defined as operation in the absence of free oxygen but in the presence of bound oxygen (e.g., nitrates, nitrites). Choosing which products to recover affects the technologies used and in their configuration (Cai et al., 2020). Biological treatment, including biological nutrient removal, is usually seen as a tertiary wastewater treatment process, reducing nitrogen and phosphorus concentrations, and producing a clean effluent suitable for discharge into receiving bodies (Wainaina et al., 2021). However, pollutants such as persistent organics (often pharmaceutical compounds) and inorganics can remain.

BNR for nitrogen recovery occurs via integrated anaerobic/aerobic/anoxic processes. Anaerobic zones are implemented initially to release N as ammonia compounds. The conventional pathway is nitrification-denitrification in which ammonia compounds are completely oxidised (e.g. in an aerobic CSTR) to produce nitrates (Reaction R3) which are then reduced to nitrogen anoxically in the presence of an electron donor (Reaction R4). Denitrification produces CO${}_{2}$ since the electron donor is usually an organic carbon compound due to the process being facilitated by a high C/N ratio.

(R3) $${\text{NH}}_{4}^{+}+2O_{2}⟶\text{NO}3^{-}+2H^{+}+H_{2}O$$

(R4) $$1.25{\text{CH}}_{2}O+{\text{NO}}_{3}^{-}+H^{+}⟶0.5N_{2}+1.25{\text{CO}}_{2}+1.75H_{2}O$$

Specifically, nitrification proceeds via the oxidation of ammonium to nitrite by ammonia oxidising biomass (AOB), followed by further oxidation to nitrate by nitrite oxidising biomass (NOB) as shown by Reactions R5 and R6, respectively.

(R5) $$\text{NH}4^{+}+1.5O_{2}⟶{\text{NO}}_{2}^{-}+2H^{+}+H_{2}O$$

(R6) $${\text{NO}}_{2}^{-}+0.5O_{2}⟶{\text{NO}}_{3}^{-}$$

For higher nitrogen concentrations (lower C/N ratio), the anammox process is typically used (Lin et al., 2016). Anammox follows a partial oxidation step to produce nitrite (Reaction R7). Specifically, single reactor high activity ammonia removal over nitrite (SHARON) is an aerobic process operating at high temperatures so as to favour the growth of AOB over NOB. At low SRT, NOB is washed out such that the ammonia is primarily oxidised to nitrite. Additionally, lower aeration hinders NOB growth since 3.42 g O${}_{2}$/g N is used to oxidise NH${}_{4}$-N to NO${}_{2}$-N with an additional 0.57 g O${}_{2}$/g N to convert the NO${}_{2}$-N to NO${}_{3}$-N (United States Environmental Protection Agency, 2010). The nitrite is subsequently used as the electron acceptor in a reduction reaction and converted to nitrogen gas by anammox bacteria under anoxic conditions (Reaction R8) (Kuenen, 2008). This pathway has two advantages over conventional nitrification-denitrification: reduced aeration costs of about 60% and no additional requirement for organic carbon dosing (Ma et al., 2018).

(R7) $$\begin{array}{ccc} & & 2{\text{NH}}_{4}^{+}+1.5O2+2{\text{HCO}}_{3}^{-}\\ & & \;⟶\\ & & {\text{NH}}_{4}^{+}+{\text{NO}}_{2}^{-}+2{\text{CO}}_{2}+3H_{2}O \end{array}$$

(R8) $${\text{NH}}_{4}^{+}+{\text{NO}}^{2-}⟶N_{2}+2H_{2}O$$

**Fig. 9:**
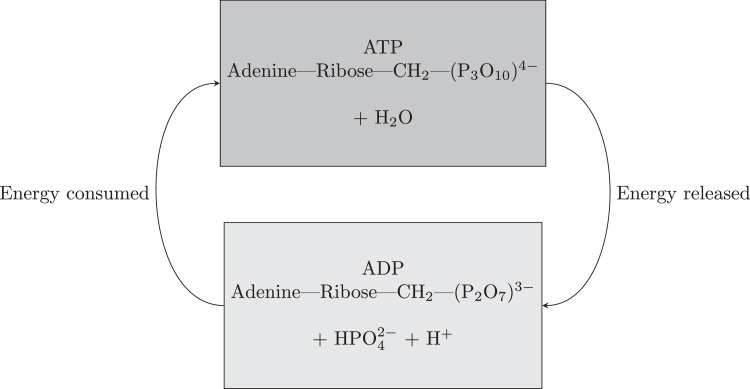
Intracellular energy transfer via adenosine triphosphate (ATP) and adenosine diphosphate (ADP).

Phosphorus can exist in wastewater as ortho-phosphate which is immediately available for biological metabolism, poly-phosphates (molecules containing 2 or more P atoms) which are hydrolysed in aqueous solutions, or organic phosphates. These phosphate molecules, store chemical potential energy within covalent bonds such as the phosphoanhydride bond. Breaking these bonds and releasing phosphate molecules also results in energy release which is the mechanism used to transfer intracellular energy via the conversion of ATP to ADP (Fig. 9).

**Fig. 10:**
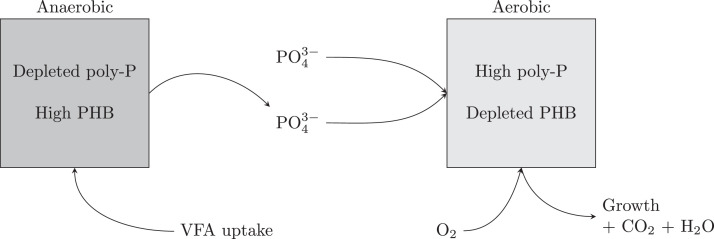
Biological phosphorus removal. Under anerobic conditions, polyphosphate (poly-P) stores are used to generate energy releasing phosphates (PO${}_{4}^{3-}$) to facilitate uptake of volatile fatty acids (VFAs) stored as polyhydroxybutyrate (PHB). Then under aerobic conditions PHB stores are metabolised in the presence of dissolved oxygen (O${}_{2}$), producing enough energy for growth as well as luxury PO${}_{4}^{3-}$ uptake which is stored as poly-P, along with byproducts carbon dioxide (CO${}_{2}$) and water (H${}_{2}$O).

In a similar pathway, phosphorus-accumulating organisms (PAOs) utilise their polyphosphate (poly-P) stores to generate energy by releasing readily available ortho-phosphates into the wastewater (Achbergerov and Nahlka, 2011). Under anaerobic conditions, this enables PAOs to continue to uptake volatile fatty acids (VFAs) e.g., acetic acid, for storage as polyhydroxyalkanoates (PHAs), most commonly polyhydroxybutyrate (PHB). This gives the PAOs a great advantage when introduced to aerobic conditions, wherein the PHB stores are metabolised with the dissolved oxygen to generate energy and uptake excess phosphorus, in the form of phosphate in the wastewater, to be stored again as poly-P (Fig. 10) (United States Environmental Protection Agency, 2010). The storage of phosphates by PAOs can also occur under anoxic conditions, whereby nitrate/nitrite is utilised as the electron acceptor (Kerrn-Jespersen and Henze, 1993). Therefore, enhanced biological P recovery (EBPR), typically implements an anaerobic process followed by anoxic/aerobic processes followed by the recovery of P-rich sludge via clarification (Raj et al., 2013). Additionally, side stream enhanced biological phosphorus removal (S2EBPR) systems adopt a fermentation reactor to enhance VFA production from the RAS before recycling to the anaerobic reactor. EBPR is sensitive to various process parameters: readily biodegradable COD to TP ratio so that there is enough fermentable substrate for the PAOs to grow; ensuring no dissolved oxygen or nitrates in the anaerobic zone; temperature; pH; process configuration; recycle flows; and reactor sizing.

Research gaps remain on biosynthesis and microbiome engineering to recover resources from FPWW which offer considerable recovery potential due to their high concentrations of organics and nutrients, and the absence of hazardous contaminants. This has been confirmed by a recent publication (Durkin et al., 2022b) which has proposed to close the loop by coupling targeted separation with microbial mono-cultural and co-cultural biosynthesis to efficiently recover the carbon and nutrients from wastewater. In particular, after high-value solutes have been recovered, a mixture of organic carbon and inorganic nutrients remain, which bacteria and yeasts can transform into valuable bio-molecules. Very little research has been reported on the biosynthesis and fermentation of mixed composition wastewater to valuable end products such as feed protein and other solutes. Thus far, fewer than 10 fungal, bacterial or microalgal strains have been identified that can produce feed-grade microbial protein from food/drink wastewaters (e.g. soya, brewing WW) at lab or commercialized scales (Jones, Karpol, Friedman, Maru, Tracy, 2020, Piercy, Verstraete, Ellis, Banks, Rockström, Smith, Witard, Hallett, Hogstrand, Knott, Karwati, Rasoarahona, Leslie, He, Guo, 2022, Ritala, Häkkinen, Toivari, Wiebe, 2017).

#### 2.5.3 Thermochemical technologies

**Fig. 11:**
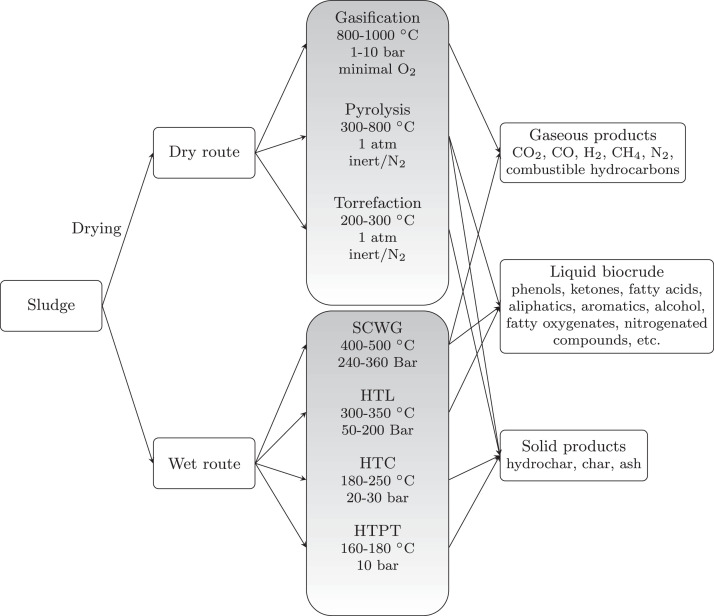
Dry and wet thermochemical processing routes for sludge. SCWG – Super Critical Water Gasification HTL – High Temperature Liquifaction HTC – High Temperature Carbonisation HTPT – High Temperature Pre-Treatment.

Thermochemical technologies use high temperatures to break long-chain molecules into short-chain molecules, creating highly calorific chemical platforms with a spectrum of end-uses, from fuels to high-value platform chemicals. While most often employed to treat sewage sludge, thermochemical processes can also be used for pretreatment (prehydrolysis) to enable increased energy or resource recovery in downstream processes (e.g., enhanced biogas production via AD (Pilli et al., 2015) or protein recovery from solubilised sludge (García et al., 2017)). Enhanced energy recoveries arise due to the decomposition of larger molecules into smaller molecules, increasing particle surface area, thereby increasing degradability. Likewise, biopolymers are released as cells release their intracellular compounds upon decomposition, making these resources more readily available for recovery. Thermochemical technologies include incineration, hydrothermal processes, torrefaction, pyrolysis and gasification, where the technologies differ in operating temperature and pressure and presence of oxygen, water or additional reactants, as shown in Fig. 11.

Thermochemical sludge treatment requires dewatering as a pre-treatment, which produces a concentrated and a dilute stream. Typically, the dilute stream is usually directed back to the inlet of the wastewater treatment works. With the exception of high temperature carbonisation, thermochemical processing does not typically produce an aqueous effluent stream requiring further treatment.

Incineration is a high temperature ($\geq \;850{\;}^{\circ }$C) combustion process with several advantages: primarily, superior sludge volume reduction producing a small amount of ash (about 10% of the volume of mechanically dewatered sludge); removal of toxic organic material and pathogens via thermal destruction; and the recovery of flue gas energy (Tang et al., 2008). However, numerous environmental concerns have been raised regarding emissions from incineration processes, including: dioxin-related substances (a class of toxic, persistent organic pollutants), NO${}_{x}$, SO${}_{2}$ and heavy metals (Dyke, Foan, Fiedler, 2003, Han, Xu, Yao, Furuuchi, Sakano, Kim, 2008, Zhou, Meng, Long, Li, Zhang, 2015). This results in high scrubbing costs of the product gas making it important to seek alternatives for thermochemical sludge remediation (Manara and Zabaniotou, 2012).

Hydrothermal processes use water at sub-critical conditions where it becomes an effective solvent and highly reactive medium with changes in solubility, density and dielectric constant (Jena et al., 2011). Processes included here are hydrothermal pretreatment (HTPT), hydrothermal carbonisation (HTC) and hydrothermal liquefaction (HTL). Whereas pyrolysis and gasification typically require an energy intensive drying step, hydrothermal technologies can process wet biomass (Wikberg et al., 2015). Full-scale HTPT at 160–180 ${}^{\circ }$C and 8–10 bar has been shown to reduce AD sludge retention time and sludge volume whilst increasing dewaterability and biogas production (Pilli et al., 2015). However, at higher temperatures (≥ 190 ${}^{\circ }$C) biogas production from subsequent AD is lower (biodegradability is reduced) due to Amadori rearrangement and Maillard reaction product formation (Danso-Boateng et al., 2015).

HTC operates at higher temperatures and pressures (180–250 ${}^{\circ }$C and 20–30 bar) than HTPT to produce three readily separable products: a solid hydrochar platform as the primary product; an aqueous byproduct containing organic and inorganic material; and a gas phase containing primarily CO${}_{2}$ (Libra et al., 2011). The carbon-rich hydrochar has various uses depending on the oxygen-carbon ratio, including: a potential fuel source, a soil conditioner (Libra et al., 2011), and a platform for biofuel, VFA or syngas production (Danso-Boateng et al., 2015). HTL processes operate at higher temperatures and pressures (300–350 ${}^{\circ }$C and 50–200 Bar) to favour the production of a viscous liquid biocrude over the solid hydrochar (Chen et al., 2014). Similarly, super critical water gasification (SCWG) operates at even higher temperatures and pressures (300–500 ${}^{\circ }$C and 240–360 Bar) favouring the production of biocrude and non-condensable gases.

Pyrolysis refers to the thermal degradation of biomass in the absence of oxygen to produce: primarily, a viscous liquid biocrude (pyrolysis oil or bio-oil) containing organic and inorganic compounds; a solid biochar containing heavy metals and inert substances; and non-condensable gases such as hydrogen, carbon oxides and methane (Sharifzadeh et al., 2019). Pyrolysis is a flexible process allowing for various process configurations and operating conditions (temperatures in the range 300–900 ${}^{\circ }$C and residence times ranging from seconds to hours) and accepting a range of feedstocks including sewage sludge, municipal solid waste (MSW), forest residue, food waste and paper waste (Werle and Wilk, 2010). Specifically, lower temperatures and longer residence times favour charcoal production whilst high temperatures and longer residence times favour a gaseous product (Bridgwater, 2012). Alternatively, fast pyrolysis favours the production of liquid products by operating at moderate temperatures (450–600 ${}^{\circ }$C) and short residence times of less than 2 seconds (Sharifzadeh et al., 2019).

Fast pyrolysis is ideal for integration with other technologies due to its shorter residence time facilitating continuous operation, scale up and commercialisation. Fluidised bed reactor configurations are leading the way in this regard offering efficient heat transfer and high bio-oil yield but are limited by the need for preprocessing of sludge to obtain fine particles (Sharifzadeh et al., 2019). Several models have been posed over the years for these reactors with fluidisation gas temperature being highlighted as the most important parameter (Lathouwers, Bellan, 2001, Trendewicz, Braun, Dutta, Ziegler, 2014).

Gasification operates at higher temperatures than pyrolysis (800–1000 ${}^{\circ }$C) in the presence of gasifying agents, such as steam and CO${}_{2}$. Gasification, through reactions with gasifying agents and partial oxidation, converts the pyrolysis reaction products (char, tars and gases) to syngas (a mixture of H${}_{2}$, CO, CH${}_{4}$ and CO${}_{2}$), which can be used to produce heat and electricity directly or converted into liquid fuels or platform chemicals (Mahinpey, Gomez, 2016, Molino, Larocca, Chianese, Musmarra, 2018). More specifically, the gasification process involves four steps: drying; pyrolysis reactions; partial oxidation to produce steam and CO${}_{2}$ for use as gasifying agents, as well as CO and heat; and reduction reactions that consume the gasifying agents and use the heat from oxidation to facilitate the endothermic reactions (You et al., 2017). The reduction reactions include steam and CO${}_{2}$ (Boudouard reaction) gasification (Reactions R9 and R10), steam and dry reformation of methane (Reactions R11 and R12), water-gas shift (Reaction R13) and methanation (Reaction R14).

(R9) $$C+H_{2}O⇌H_{2}+\text{CO}$$

(R10) $$C+{\text{CO}}_{2}⇌2\text{CO}$$

(R11) $${\text{CH}}_{4}+H_{2}O⇌\text{CO}+3H_{2}$$

(R12) $${\text{CH}}_{4}+{\text{CO}}_{2}⇌2\text{CO}+2H_{2}$$

(R13) $$\text{CO}+H_{2}O⇌{\text{CO}}_{2}+H_{2}$$

(R14) $$C+2H_{2}⇌{\text{CH}}_{4}$$

Reactor configurations for gasification include fixed bed, fluidised bed and entrained flow reactors, with other important process parameters including temperature, residence time, heating rate, feedstock surface area and alkali content (You et al., 2017).

### 2.6 Barriers to technology deployment

There exist multiple recent reviews in the literature on the topic of resource recovery from wastewater (Asgharnejad, Khorshidi Nazloo, Madani Larijani, Hajinajaf, Rashidi, 2021, Costa, Amorim, Duque, Reis, Castro, 2022, Diaz-Elsayed, Rezaei, Guo, Mohebbi, Zhang, 2019, Diaz-Elsayed, Rezaei, Ndiaye, Zhang, 2020, Durkin, Guo, Wuertz, Stuckey, 2022, Guerra-Rodríguez, Oulego, Rodríguez, Singh, Rodríguez-Chueca, 2020, Kundu, Dutta, Samanta, Dey, Sherpa, Kumar, Dubey, 2022, Puyol, Batstone, Hülsen, Astals, Peces, Krömer, 2017, Renfrew, Vasilaki, McLeod, Lake, Danishvar, Katsou, 2022, Solon, Volcke, Spérandio, Loosdrecht, 2019, Tóth, Fózer, Mizsey, Varbanov, Klemeš, 2022, Yadav, Mishra, Ghosh, Sindhu, Vinayak, Pugazhendhi, 2021). Whilst the majority of these reviews focus on resource recovery from municipal wastewater, applications to the food industry, the wastewater of which contains significant amounts of recoverable energy, nutrients, and value-added products, are comparatively lacking (Angenent, Karim, Al-Dahhan, Wrenn, DommÃguez-Espinosa, 2004, Asgharnejad, Khorshidi Nazloo, Madani Larijani, Hajinajaf, Rashidi, 2021, Costa, Amorim, Duque, Reis, Castro, 2022, Durkin, Guo, Wuertz, Stuckey, 2022). However, realising the potential of resource recovery from FPWW requires integrated decision-making processes considering economic, environmental, and social criteria (as well as practical operability and process resilience) across multiple life cycle stages (Asgharnejad, Khorshidi Nazloo, Madani Larijani, Hajinajaf, Rashidi, 2021, Byrne, Lohman, Cook, Peters, Guest, 2017, Costa, Amorim, Duque, Reis, Castro, 2022, Diaz-Elsayed, Rezaei, Guo, Mohebbi, Zhang, 2019, Diaz-Elsayed, Rezaei, Ndiaye, Zhang, 2020, Durkin, Guo, Wuertz, Stuckey, 2022, Gherghel, Teodosiu, Notarnicola, De Gisi, 2020, Juan-García, Butler, Comas, Darch, Sweetapple, Thornton, Corominas, 2017, Kundu, Dutta, Samanta, Dey, Sherpa, Kumar, Dubey, 2022, Renfrew, Vasilaki, McLeod, Lake, Danishvar, Katsou, 2022, Solon, Volcke, Spérandio, Loosdrecht, 2019, Tóth, Fózer, Mizsey, Varbanov, Klemeš, 2022, Villarín, Merel, 2020, Yadav, Mishra, Ghosh, Sindhu, Vinayak, Pugazhendhi, 2021). Other challenges cited as constraining resource recovery from wastewater include: the high cost of pilot studies and demonstrative plants (Diaz-Elsayed, Rezaei, Guo, Mohebbi, Zhang, 2019, Puyol, Batstone, Hülsen, Astals, Peces, Krömer, 2017, Yadav, Mishra, Ghosh, Sindhu, Vinayak, Pugazhendhi, 2021); the variability and uncertainties in wastewater quantity and quality (Asgharnejad, Khorshidi Nazloo, Madani Larijani, Hajinajaf, Rashidi, 2021, Costa, Amorim, Duque, Reis, Castro, 2022, Durkin, Guo, Wuertz, Stuckey, 2022, Tóth, Fózer, Mizsey, Varbanov, Klemeš, 2022); the difficulties of wastewater characterisation to determine resource availability for recovery (Asgharnejad, Khorshidi Nazloo, Madani Larijani, Hajinajaf, Rashidi, 2021, Durkin, Guo, Wuertz, Stuckey, 2022, Tóth, Fózer, Mizsey, Varbanov, Klemeš, 2022, Villarín, Merel, 2020); public opinion, company culture, and the “yuck factor” towards recovering resources from waste (Costa, Amorim, Duque, Reis, Castro, 2022, Guerra-Rodríguez, Oulego, Rodríguez, Singh, Rodríguez-Chueca, 2020, Villarín, Merel, 2020); the difficulty of model development for resource recovery processes (Durkin, Guo, Wuertz, Stuckey, 2022, Solon, Volcke, Spérandio, Loosdrecht, 2019); and the lack of retrofit/integrated process design frameworks to select from multiple technologies and multiple resources to be recovered (Diaz-Elsayed, Rezaei, Ndiaye, Zhang, 2020, Durkin, Guo, Wuertz, Stuckey, 2022, Guerra-Rodríguez, Oulego, Rodríguez, Singh, Rodríguez-Chueca, 2020, Yadav, Mishra, Ghosh, Sindhu, Vinayak, Pugazhendhi, 2021).

Despite advances in resource recovery technology (Puyol et al., 2017), its use is limited within industry. This is due to uncertainties in economic and environmental performance at industrial scale compared to lower-risk traditional wastewater treatment options (Sanford et al., 2016), and limitations in company culture (Kirchherr et al., 2018). Under a paradigm shift towards wastewater-to-resource “biorefining” (Chen et al., 2018), wastewater is considered as a source of various fractions which can be separated and further processed to value-added products, akin to crude oil processing within petroleum refineries. It has been shown that implementing such organic waste biorefineries is constrained by uncertainties in waste composition and availability (Alibardi et al., 2020). Similarly, resource recovery from FPWW depends on highly variable FPWW volumes and compositions due to batch processing and changes in operating variables.

A number of potential reaction pathways underpin resource recovery from FPWW, and process synthesis and design is subject to multiple design criteria. Resource recovery can be regarded as a system of resource flows and reaction pathways that are subject to complexity, nonlinearity, and uncertainty. The deployment and integration of new resource recovery technologies into biorefineries necessitates holistic systems-wide analyses based on computational modelling, simulations, and mathematical optimisation to determine the economic, environmental, and operational trade-offs during early-stage process synthesis and design (Diaz-Elsayed et al., 2019). However, the optimisation of resource recovery from FPWW to maximise economic return, while minimising environmental damage still remains very underdeveloped, and very little has been published in this area. This would require the capacity to integrate multiple disciplines to develop fully integrated process systems (Durkin et al., 2022b). Whilst individually many technologies are fully mature and in commercial use, the integration of multiple technologies within the same process requires additional developments in computational design tools and optimisation methodologies.

## 3 Process systems engineering for resource recovery

As a discipline, PSE is well placed to harness recent developments in AI (Qin and Chiang, 2019) to address the challenges of optimising sustainable systems for resource recovery within a circular economy (Pistikopoulos et al., 2021). According to Sargent, the father of PSE, “Process systems engineering concerns the development of systematic techniques for process modelling, design and control... some formulate their synthesis, design and/or control problem, or some useful simplification of it, in precise mathematical terms, and then seek to exploit the mathematical structure to obtain an effective algorithm” (Sargent, 1983). In this regard, AI techniques, particularly machine learning (ML) methods (Schweidtmann et al., 2021), can be used to assist in the mathematical formulation and simplification of the resource recovery process synthesis and design problem (Schneider et al., 2022).

The synthesis and design of integrated resource recovery processes, also referred to as biorefineries, is widely applied to the valorisation of organic solid wastes, but applications to resource recovery from wastewater are relatively lacking (Alibardi, Astrup, Asunis, Clarke, De Gioannis, Dessi, Lens, Lavagnolo, Lombardi, Muntoni, Pivato, Polettini, Pomi, Rossi, Spagni, Spiga, 2020, Chen, Ho, Nagarajan, Ren, Chang, 2018, Cherubini, Jungmeier, Wellisch, Willke, Skiadas, Van Ree, de Jong, 2009, Gong, You, 2015, Gong, You, 2015, Kumar, Samadder, 2017, Leong, Chang, Khoo, Chew, Chia, Lim, Chang, Show, 2021, Pott, Johnstone-Robertson, Verster, Rumjeet, Nkadimeng, Raper, Rademeyer, Harrison, 2018, Rajesh Banu, Kavitha, Yukesh Kannah, Bhosale, Kumar, 2020). However, the literature on organic solid waste biorefineries provides a perspective on the challenges facing integrative process design for resource recovery from wastewater. Specifically, whilst biorefinery concepts provide a computational framework for early-stage integrative process design, more granular challenges arise during implementation, for example: mathematical modelling of resource recovery process units (Puchongkawarin et al., 2015); computational tractability of modelling and optimising integrative superstructures (Alva-Argáez, Kokossis, Smith, 1998, Gong, You, 2015); methods to hedge against process and model uncertainties (Gong and You, 2015a); and integrated decision-making and the corresponding definition of optimality (Gong, You, 2015, Gong, You, 2015).

Computer-aided process synthesis methods can be used to screen alternative process networks without the time and capital expenditures required for pilot studies (Chen, Grossmann, 2017, Yeomans, Grossmann, 1999). These methods have been widely applied to the design of wastewater treatment plants (Ahmetović, Grossmann, 2011, Al, Behera, Gernaey, Sin, 2020, Bozkurt, van Loosdrecht, Gernaey, Sin, 2016, Bozkurt, Quaglia, Gernaey, Sin, 2015, Castillo, Cheali, Gómez, Comas, Poch, Sin, 2016, Padrón-Páez, Almaraz, Román-Martinez, 2020), whilst applications to integrative processes for resource recovery from wastewater remain relatively unexplored (Durkin, Finnigan, Johnson, Kazer, Yu, Stuckey, Guo, 2022, Juznic-Zonta, Guisasola, Baeza, 2022, Puchongkawarin, Gomez-Mont, Stuckey, Chachuat, 2015). This research gap is constricted by the complexity of modelling resource recovery processes in the context of wastewater systems (Puchongkawarin et al., 2015).

Mathematical modelling is an indispensable tool for designing the resource recovery processes within a circular economy and sustainable future (Schneider et al., 2022). Specifically, wastewater treatment plant design has undergone a paradigm shift in the last decades from a dependence on expert knowledge and well-established guidelines, towards sophisticated modelling and simulation technologies (Al et al., 2020). This transition was made possible by the development of rigorous mathematical models of wastewater systems, based on first-principles physics, chemistry, and biology, including models for anaerobic digestion (Batstone et al., 2002a) and activated sludge processes (Henze et al., 2006). However, these models tend to be highly dimensional and complex, because of the biological nature of the underlying processes. These models can become particularly cumbersome during equation-oriented integrated process design, wherein the mathematical formulations are coded directly into specialist optimisation software, due to the combinatorial nature of modelling superstructures comprising many possible reaction pathways (Alva-Argáez et al., 1998). However, the continued development of rigorous models for emerging resource recovery technologies is important as these models form the basis for all other modelling methods discussed in this work (by providing a source of high-fidelity data from process simulations) as well as providing validation for more computationally tractable approximations (Solon et al., 2019).

There exists 3 approaches to address the computational intractability of incorporating rigorous models directly within equation-oriented decision-making frameworks. First, lower-fidelity short-cut models can be formulated as more computationally tractable approximations to rigorous models whilst maintaining their foundations in first-principles (Razavi et al., 2012). (Bozkurt, van Loosdrecht, Gernaey, Sin, 2016, Bozkurt, Quaglia, Gernaey, Sin, 2015, Castillo, Cheali, Gómez, Comas, Poch, Sin, 2016) utilise lower-fidelity general models to optimise municipal wastewater treatment plants, including validation against rigorous simulation-based models. Similarly, there exists significant applications of optimisation methodologies to water networks wherein short-cut models are utilised to represent water and contaminant flows with simple conversion factors (Ahmetović, Grossmann, 2011, Alva-Argáez, Kokossis, Smith, 1998).

A second option is to utilise design of experiments (Sacks et al., 1989) and process simulation software, embedding rigorous models within sequential modular black boxes along with algorithms to enable solution convergence for complex process networks. Simulation software is widely used to evaluate process design performance within a broad range of applications including prediction, design, operation, sensitivity analysis, and optimisation (Razavi et al., 2012). However, the requirement for more accurate process models necessitates a large number of simulation evaluations resulting in excessively high computational costs. Additionally, the underlying code within process simulation software is often embedded as black box models where functional and derivative information is unavailable to the user (Amaran et al., 2016).

Thirdly, increased data availability and computational efficiency has driven recent advances in data-driven AI and ML models applied to wastewater systems for sustainable decision-making (Hadjimichael et al., 2016), process analysis, operation, and control (Corominas, Garrido-Baserba, Villez, Olsson, Cortés, Poch, 2018, Newhart, Holloway, Hering, Cath, 2019), prediction, classification, water quality evaluation (Zhu et al., 2022), uncertainty analysis, and optimisation (Schneider et al., 2022). These data-driven models are effective at modelling nonlinear wastewater systems (Zhu et al., 2022) but depend highly on the quantity and the quality of data used to train them (Dobbelaere, Plehiers, Van de Vijver, Stevens, Van Geem, 2021, Schneider, Quaghebeur, Borzooei, Froemelt, Li, Saagi, Wade, Zhu, Torfs, 2022). Additionally, process simulation software can be used to generate large, high-fidelity data from first-principles models with which to train representative ML models as surrogates (Bradley et al., 2022). However, a division between traditional engineering specialists and computer scientists limits the development and deployment of such data-driven decision-support tools to complex industrial problems (Corominas, Garrido-Baserba, Villez, Olsson, Cortés, Poch, 2018, Dobbelaere, Plehiers, Van de Vijver, Stevens, Van Geem, 2021, Hadjimichael, Comas, Corominas, 2016, Himmelblau, 2008, Newhart, Holloway, Hering, Cath, 2019).

Two branches of AI that are of particular interest for process synthesis and optimisation are the fields of ML and derivative-free optimisation (DFO). ML models provide a data-driven approach to represent resource recovery processes within larger decision-making frameworks (Wei, 2013). DFO refers to algorithms used to solve black box optimisation (BBO) problems without derivative information (Audet and Hare, 2017). Different branches of DFO can be categorised dependent on whether surrogate ML models are used to guide the search (model-based DFO) or whether the data is examined directly for improvements in optimality (direct-search DFO) (Bhosekar and Ierapetritou, 2018a). Additionally, Bayesian DFO methods exist at the interface of these two approaches: following a direct-search approach of directly analysing data for optimality whilst simultaneously harnessing features of surrogate modelling components within optimised acquisition functions (Frazier, 2018).

This literature review proceeds with a general background on mathematical optimisation as it is an important part of the PSE methods underpinning this work. This also provides background for specific branches of optimisation modelling such as superstructure optimisation applied to solve process synthesis problems, and BBO and DFO techniques to harness first-principles models within process simulation software.

### 3.1 Holistic process design

Holistic process design methods couple systems analyses with early-stage design and optimisation frameworks to determine sustainably optimal designs for economic and environmental objectives (Solon et al., 2019). Chemical process and wastewater treatment simulators can be used to model full-scale resource recovery. LCA can be further coupled with such simulators to evaluate the environmental sustainability of scaled-up technologies. Then, mathematical optimisation, using specialised programming languages (e.g., GAMS, Pyomo), enable decision-makers to choose from the many different resource recovery technologies and reaction pathways (Baratsas, Pistikopoulos, Avraamidou, 2021, Puchongkawarin, Mattaraj, 2020, Somoza-Tornos, Pozo, Graells, Espuña, Puigjaner, 2021). These are often posed as MIP problems, where binary variables represent the activation/deactivation of competing resource recovery processing routes. Significant influences of uncertainties on integrative resource recovery process economic performance have been observed in a MILP study on wastewaters (Puchongkawarin and Mattaraj, 2020). Multi-objective decision-making, and the trade-off between economic profit and environmental life cycle impacts, are also addressed in previous mathematical programming research (Baratsas, Pistikopoulos, Avraamidou, 2021, Somoza-Tornos, Pozo, Graells, Espuña, Puigjaner, 2021).

Multi-objective optimisation (MOO) provides a PSE approach to holistic process synthesis by enabling the trade-off between multiple decision criteria to be systematically optimised. A Pareto-optimal frontier is defined by a set of non-dominated solutions, such that no objective can be improved without sacrificing optimality in other objectives. Non-dominated solutions therefore sit along the Pareto-optimal frontier, which lies on the boundary in performance space between feasible and infeasible solutions. MOO has been widely applied to many process design applications, particularly in studies focused on sustainable process design (Gassner, Maréchal, 2009, Gong, You, 2015, Gonzalez-Garay, Guillen-Gosalbez, 2018, Grossmann, Guillén-Gosálbez, 2010, Guillén-Gosálbez, 2011, Hennen, Postels, Voll, Lampe, Bardow, 2017). However, applications to wastewater treatment, particularly with a focus on resource recovery, are comparatively lacking.

One method to determine the economic performance of resource recovery systems is techno-economic assessment (TEA), where empirical data is used to determine capital and operating expenditures during screening of process design alternatives. Reviews of TEA and systems-wide analyses for bioproduct production which could be applied to the recovery of such products from FPWWs exist in the literature (Scown et al., 2021). Commercial software (Aspen, SuperPro, GPS-X) enables users to build bio-based processes models and perform TEA, but it is also possible to develop spreadsheet-based methods to cost these processes. TEA can also be used to determine economic “pinch points” (Zagklis et al., 2021) and inform cost-benefit analyses (Pandey et al., 2020) of such integrated processes.

Whilst traditional process design would optimise economic performance with no regard for the environment, more recently businesses have begun to optimise economic performance under environmental constraints. As part of the transition to more sustainable economic practices, in the medium term, process design should incorporate both economic and environmental indicators into the optimisation objective function, whilst in the long term, perhaps environmental objectives should be optimised under financial constraints (Schoenmaker, 2019). Carbon pricing is an established financial instrument used to impose the external costs of greenhouse gas emissions on the emitters instead of the public. Recent work has determined the impact of carbon pricing, beyond GHG emissions and climate change, on other Earth system processes and planetary boundaries (PBs) (Engström et al., 2020).

Life cycle assessment (LCA) is a widely adopted methodology for determining environmental impacts across an entire life cycle. Like TEA, process simulation tools can be used to generate input-output data for analysis in LCA tools (SimaPro, OpenLCA). A recent review (Corominas et al., 2013) of LCA and its application to wastewater treatment highlights that better integration with design tools is necessary to inform decision-makers. Chen et al. (Chen et al., 2020) presented a hybrid LCA, where the life cycle inventory was obtained from a combination of bottom-up (process-based) and top-down approaches (macroeconomics-based), for resource recovery from FPWW. Other recent LCA studies were also performed to analyse the SCP grown on potato wastewater (Spiller et al., 2020), and hydrogen recovery from food waste (Sarkar et al., 2021).

As discussed in the introduction, while conventional LCA can only be used to assess the relative sustainability of different solution across different environmental metrics, PB-LCA can be used to provide an absolute indication of sustainability. This can correspond to moving from a multi-objective optimisation, with each indicator an objective in its own right, to a constrained single objective optimisation, in which each of the PB must be respected. In many cases, maximising profit, or minimising cost would remain in the objective function. Of course, setting numerical values for these constraints depends on appropriate down-scaling of the PBs, as discussed in Section 1.3.

Holistic modelling approaches covering treated effluent qualities, recovered product specifications, techno-economic concepts and life cycle costs and emissions are required. More modelling frameworks focusing on operability in practical operating environments are needed to ensure modelled process performance is realisable at full-scale and under real-world uncertainties. Such problems can be addressed by methods for optimisation under uncertainty such as stochastic optimisation, robust optimisation, and flexibility analysis. Additionally, the competing interests of multiple stakeholders could be addressed by the application of game theory concepts. Such methods can highlight commercial business opportunities, promising technologies, and processes to be financially incentivised by policy decision-makers for the realisation of a circular economy (Baratsas, Pistikopoulos, Avraamidou, 2021, Somoza-Tornos, Pozo, Graells, Espuña, Puigjaner, 2021).

Some challenges with solving holistic MOO problems, particularly as the number of objectives increase, are: increased dimensionality of the Pareto front; increased computational costs; difficulties in visualising the objective space; and in the case where heuristic-based search algorithms are used, stagnation of search processes can occur (Deb and Saxena, 2005). The latter occurs as search algorithms favour non-dominated solutions within a population, putting more emphasis on them, and thereby not searching for other non-dominated solutions that might exist (Deb and Saxena, 2005).

Another challenge with MOO is how to enumerate the trade-offs between different objectives so that a single solution can be found. There are many methods to deal with this challenge including: scalarisation techniques such as weighted sum or ε-constraint methods; involving decision makers to define reduced search spaces; finding “knee” solutions where moving from one solution to a neighbouring one requires a large sacrifice in at least one objective for a relatively small gain in another; finding robust frontiers where solutions are least sensitive to parameter uncertainties; and eliminating redundant objectives (Deb and Saxena, 2005). The method of removing redundant objectives has generated a lot of research interest as a systematic method to reduce objective space dimensionality, thereby tackling the root of all the challenges mentioned above. Objectives can be removed using principal component analysis (PCA) (Deb and Saxena, 2005), or by minimisation of the delta error, which removes objectives without loss of information that would alter the solution dominance structure (Brockhoff, Zitzler, 2006, Guillén-Gosálbez, 2011).

### 3.2 Model validation & regularisation

“All models are wrong. Some are useful.”

George Box, British Statistician.

Model validation is part of the process of discerning useful models from less useful ones. This is the process of ensuring that the modelling errors introduced by a model are acceptable in the context of the decisions the model is being used to inform (Bhosekar and Ierapetritou, 2018b). Validation is carried out by evaluating the error between predictions from the model using identified parameter values and observations from the ground truth as reserved testing inputs. These errors are then used to calculate model validation metrics to determine the accuracy of the model. Some common validation metrics used for regression models are shown in Table 5. These validation metrics are also often embedded within training algorithms that minimise the model errors thereby improving the model fit. If a surrogate model is being used to approximate a high-quality simulator, the simulator will be used as the source of ground truth. If a model is being used to approximate a real system or process, process data should be used as the ground truth.

**Table 5:** Regression model validation metrics.

Metric	Formula
Mean absolute error	$\frac{1}{n}\sum_{i}\left|\epsilon_{i}\right|$
Mean squared error	$\frac{1}{n}\sum_{i}\epsilon_{i}^{2}$
Mean squared logarithmic error	$\frac{1}{n}\sum_{i}{\left(\frac{\log(1+f\left(x_{i}\right))}{\log(1+\hat{f}\left(x_{i}\right))}\right)}^{2}$
Mean absolute percentage error	$\frac{1}{n}\sum_{i}\frac{\left|\epsilon_{i}\right|}{\max\left(\omega ,\left|\hat{f}\left(x_{i}\right)\right|\right)}$
Median absolute error	$\text{median}\left(|\epsilon_{i}|\right)$
Explained variance	$1-\frac{\sum_{i}{\left(\epsilon_{i}-\mu_{\epsilon }\right)}^{2}}{\sum_{i}{\left(\hat{f}\left(x_{i}\right)-\mu_{\hat{f}}\right)}^{2}}$
Coefficient of determination, R${}^{2}$	$1-\frac{\sum_{i}\epsilon_{i}^{2}}{\sum_{i}{\left(\hat{f}\left(x_{i}\right)-\mu_{\hat{f}}\right)}^{2}}$
Maximum absolute error	$\max_{i}\left(\right|\epsilon_{i}\left|\right)$

Over-fitting occurs when over-parameterised models are fit too closely to training data such that they lose their generality and predictive performance on interpolated and extrapolated data. Specifically, over-fitted models interpret noise in the training data as features of the underlying model or system, and so it is subsequently incorporated into the model, resulting in increased model variance, meaning that the differences between models trained on different sub-sets of the training data are larger. The trade-off is that over-fitted models can achieve low model bias, which refers to the difference between the model predictions and the training data. There therefore exists a bias-variance trade-off wherein both the bias and variance should be minimised to ensure a good model fit to the training data whilst reducing the error between predictions and new observed data. Regularisation is a method to combat over-fitting by incorporating penalty terms into training loss functions that penalise model complexity and so increase model generality. Regularisation therefore increases model bias in order to drive a reduction in the model variance.

Given the inherent uncertainties and complexities of FPWW systems, incorporating model validation and uncertainty quantification (UQ) when developing surrogate models for wastewater resource recovery processes is essential for ensuring the reliability and applicability of these models in real-world scenarios. Surrogate models can significantly enhance computational efficiency and aid decision-making, but the validity of a model in a new context may not always be apparent. Whereas first-principles models are derived from explicit starting assumptions, in surrogate models these assumptions will be implicit in the training data used. The surrogate model will generally only be valid when the region of interest is similar to that of the training data. Several approaches to testing this exist. Cross-validation, in which the model is tested on a separate dataset not used during training, can be used to test the accuracy of a model with unseen data (Maremane et al., 2024). Alternatively, an applicability domain approach can be used to determine whether a surrogate model is likely to be valid (Hanser et al., 2019). Definitions of applicability can be based on the distance between the test input and the nearest training data point, or whether the test input is within a convex hull defined by the training data (Renaud et al., 2022).

Uncertainty quantification complements model validation by estimating the uncertainty in model predictions. Model uncertainty can be estimated either by using an inherently probabilistic predictive model, such as gaussian processes, or by using an ensemble model, with different models trained in different ways, or on different subsets of the data. However, each of these approaches has trade-offs associated with it: gaussian processes scale badly with large datasets (Liu et al., 2020), and the uncertainty estimates of ensemble models are not well-understood - it can be unclear when ensemble uncertainty estimates can be relied on Abdar et al. (2021). In addition to model uncertainty, it is important to consider the effect of uncertainties in the influent to a FPWW plant. Techniques such as Monte Carlo simulations can be employed to propagate uncertainties through the model, providing a probabilistic assessment of the model outputs (Kehrein et al., 2020). Integrating UQ into surrogate model development can enhance the robustness of decision-making processes in wastewater resource recovery, better allowing stakeholders to understand the limits of models’ predictions.

### 3.3 Mathematical optimisation

Mathematical optimisation has a long history of applications in PSE, with one of the earliest applications of linear programming (LP) at Gulf Oil in 1952, followed 9 years later by the first branch and bound code for solving mixed-integer linear programs (MILP) developed at British Petroleum. Over the last 70 years, the applications of mathematical optimisation to PSE have expanded to include the design and synthesis of heat exchanger networks, separations, reactors, and entire flowsheets. Temporal considerations have been formulated to enable optimised planning and scheduling, supply chains, and operation and control of process systems in real-time (Grossmann, 2021).

Mathematical optimisation problems can be formulated and solved to assist in finding optimum solutions to complex decision-making problems. Optimisation formulations are composed of an objective function to be optimised subject to a set of constraints. The objective function and constraints are functions comprising variables and parameters, where the former can be manipulated to increase or decrease the objective function and constraint values and the latter are non-varying constants. The variables and parameters can be defined over sets using index notation. Finally, the variables can be continuous and/or discrete resulting in the general optimisation formulation shown by Equations 1a–1b.



where f represents the objective function and g represents a set of constraints, which can each be function of continuous and discrete variables represented by x and y, respectively. The continuous variables are typically constrained within lower, upper real number bounds whilst discrete variables are typically binary values or other integer sets of specified dimensionalities. Generally, equality constraints can be written as two inequality constraints. Finally, due to the definition of convexity and its importance in solving optimisation problems, optimisation problems are conventionally posed as minimisation problems.

Different classes of optimisation problems depend on whether the variables include integer variables or only continuous variables and the complexity of the functions (i.e., linear, quadratic, nonlinear). The simplest category of optimisation problems fall into the class of LP. A problem that contains continuous and integer variables falls into the broad class of mixed-integer programming (MIP), whilst a problem that is linear and contains integer variables is a mixed-integer linear programming (MILP) problem. When at least one constraint or objective function is nonlinear, this results in a nonlinear programming (NLP) problem, where a sub-category exists for quadratic programming (QP). Finally, if a formulation contains both integer variables and nonlinearities, this results in a mixed-integer nonlinear programming (MINLP) problem.

Mathematical optimisation problems can be coded using state of the art optimisation software (GAMS (Bussieck and Meeraus, 2004), Pyomo (Bynum et al., 2021), JuMP (Dunning et al., 2017)) and solved by calling open-source (GLPK, IPOPT (Wachter and Biegler, 2006)) or commercial solvers (CPLEX, BARON (Sahinidis, 1996), Gurobi (Gurobi Optimization, LLC, 2022)). Formulating optimisation problems involves declaring the modelling objects (sets, parameters, variables, constraints, and objective function) and applying an appropriate solver software (depending on the class of problem i.e., LP, NLP, MILP, or MINLP) or solution algorithm to obtain solutions. Additionally, solvers can be categorised as either deterministic or metaheuristic, where the former offers guarantees of convergence to a global optimum for convex problems and the latter do not provide this guarantee in finite time (Chen and Grossmann, 2017).

### 3.4 Process synthesis

Process systems transform material and energy inputs into desired outputs via physical and chemical (and increasingly biochemical and thermochemical) transformations. Process systems design (or process synthesis) concerns the selection and assembly of process unit technologies into a process network, as well as the design parameters of the process units themselves, with the goal of optimising economic and/or environmental (and/or increasingly social) objectives (Chen and Grossmann, 2017). The process synthesis problem is addressed during the early stages of design, using computational methods to evaluate different process networks without the time and capital expenditures required for experimental and pilot studies.

A wide range of resource recovery technologies has been introduced, demonstrating that the bottleneck to process deployment is not a lack of available technologies, but a lack of decision making tools on how to integrate these technologies into an optimal flowsheet. This process synthesis problem is largely addressed by computer-aided process design whereby evaluation of different flowsheets can be achieved without the time and capital expenditures required for experimental or pilot-scale studies. Different methods for computer-aided process synthesis can be categorised into two groups: decomposition techniques and optimisation methods (Chen and Grossmann, 2017).

#### 3.4.1 Decomposition methods

Decomposition techniques involve sequentially designing each aspect of the flowsheet based on heuristics or rules-of-thumb, and provides a quick and relatively easy solution, albeit without guarantees of global optimality (Westerberg, 2004). As an example of this approach, Douglas (1985) decomposes the flowsheet synthesis problem into 5 stages: (1) continuous versus batch process selection; (2) assessing process viability based on input-output evaluation; (3) designing reactors and recycle loops; (4) design of the separation trains; (5) heat integration. Computer-aided design is used in this conceptual framework to model the reactors and separation units and evaluate different configurations.

Decomposition methods (Douglas, 1985, Siirola, Rudd, 1971) involve sequentially designing each aspect of the process system based on heuristics and engineering judgement. These decomposition techniques begin with overarching decisions about the system, such as continuous or batch processing, and increase in granularity to the detailed design of reactors, separation sequences, and heat integration. Such methods are usually supported by computational process synthesis tools, also known as flowsheeting tools, such as Aspen HSYS. Decomposing the design of process systems into a set of hierarchical decisions provides quick solutions and is relatively easy to implement (Henao and Maravelias, 2011). On the other hand, a disadvantage of these methods is that they do not ensure optimal design of the system as a whole due to the isolated and heuristic design of each sequential stage (Westerberg, 2004).

#### 3.4.2 Superstructure optimisation

Optimisation-based process synthesis methods address the shortcomings of decomposition methods by rigorously searching the design space for the process network that optimises the performance criteria represented by the objective function (Chen and Grossmann, 2017). Optimisation-based process synthesis is known as superstructure optimisation (Papoulias and Grossmann, 1983) and is a well established methodology used in many process design applications including: heat exchanger networks (Yeomans and Grossmann, 1999); energy generation and distribution (Gong, You, 2015, Yang, Zhang, Xiao, 2015); chemical processes (Gonzalez-Garay, Guillen-Gosalbez, 2018, Henao, Maravelias, 2011, Papalexandri, Pistikopoulos, 1996, Wu, Henao, Maravelias, 2016); bio-separation sequences (Wu et al., 2016); biorefining (Gassner, Maréchal, 2009, Gong, You, 2015); process water networks (Ahmetović, Grossmann, 2011, Alva-Argáez, Kokossis, Smith, 1998, Gunaratnam, Alva-Argáez, Kokossis, Kim, Smith, 2005, Karuppiah, Grossmann, 2006); and wastewater treatment (Bozkurt, van Loosdrecht, Gernaey, Sin, 2016, Castillo, Cheali, Gómez, Comas, Poch, Sin, 2016, Puchongkawarin, Gomez-Mont, Stuckey, Chachuat, 2015). However, challenges with superstructure optimisation include: postulation of the superstructure design space containing all possible configurations and interconnections between candidate process unit technologies; mathematical representation of process units within the superstructure; formulation of the process synthesis problem as a tractable MIP problem wherein integer variables are used to represent the activation/deactivation of process unit connections within the superstructure; and solving the optimisation problem for the globally optimum process system within reasonable computational time (Chen and Grossmann, 2017).

Process synthesis problems are inherently MIP problems containing both integer decision variables (for the selection of the optimal pathways through the superstructure) and continuous decision variables (for the optimisation of the selected process units). A challenge that arises when applying modelling methods to such problems is how to represent these discrete decisions within the optimisation framework. At the computer experiment stage, a challenge is how to obtain an equal representation of the discontinuous design space, or indeed if an equal distribution of samples across discrete decisions is better than a weighted approach. At the surrogate modelling and optimisation stages, one must choose whether to embed the discontinuities within the surrogate model itself, then search for a global solution using this single model or to adopt a more modular approach in which individual surrogate models are fitted to each process unit then spliced together within the optimisation problem using logic constraints (Kim and Boukouvala, 2020). Additionally, it is possible to formulate a surrogate model to represent each discrete combinatorial pathway though the superstructure – this approach is beneficial when recycle steams exist or when the definitions of interconnecting streams are highly dimensional.

**Fig. 12:**
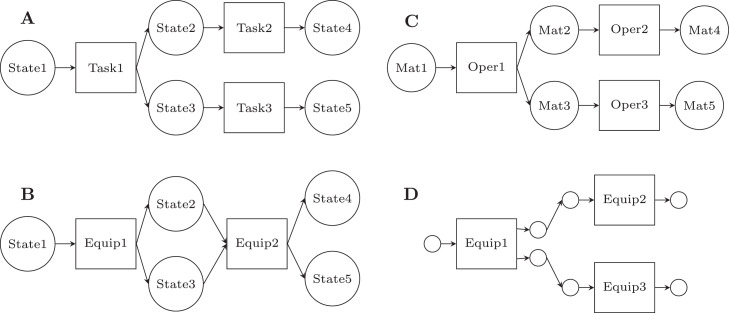
Superstructure representation approaches from Wu et al. (2016): (A) STN; (B) SEN; (C) P-graph; (D) R-graph.

The formulation of the superstructure is an important step since the solution of the optimisation problem can only exist within the postulated design space (Henao and Maravelias, 2011). The two most common superstructure representations are the state-task network (STN) (Kondili et al., 1993) and state-equipment network (SEN) (Smith and Pantelides, 1995) in which states, tasks, and equipment are used to systematically represent the superstructure (Fig. 12A and B, respectively). Specifically, states are nodes of the network defined by the set of physico-chemical properties that represent unique feeds, intermediates, and final products. Tasks are network nodes representing process operations which transforms one or more input states to one or more output states (Kondili et al., 1993). Equipment is defined in the SEN framework as the physical process units in which one or more input states can be transformed into one or more output states, via one or more tasks (Smith and Pantelides, 1995). Variations of these representations exist, such as one task one equipment STNs (STN-OTOE) where one task is allocated to one equipment unit (Yeomans and Grossmann, 1999). Other alternative superstructure representations are P-graph (Friedler et al., 1992) and R-graph (Farkas et al., 2005) representations, where the former comprises material and operation nodes, similar to STN (Fig. 12C and D, respectively). The latter considers as nodes inlet and outlet ports from process units which also act as mixers and splitters respectively. In such ways, it is possible to systematically represent a superstructure as a generalised network, thereby facilitating the formulation of a representative MIP problem.

Practically, superstructure formulation is achieved through mathematical constraints representing allowable connections between nodes of the network. A fully connected superstructure considers all possible connections thereby maximising the size of the design space. On the other hand, imposing connectivity constraints enables the removal of process network configurations that are infeasible, inferred from prior/expert/domain knowledge, or feasible configurations that are not to be considered for other reasons. Connectivity constraints can be introduced to ensure single-choice interconnection nodes for which only one outlet stream from a splitter is chosen to be nonzero (Kocis and Grossmann, 1989) or to ensure no components are fed to process units in which they should not be present (Wu et al., 2016). The former can be implemented with logic constraints on binary variables, whereas more complex rules like the latter can be implemented by defining subsets within the program.

Superstructure optimisation formulations typically implement equality constraints including material and energy balances around process units, whilst inequality constraints are used to enforce feasibility constraints and product specifications. Superstructure connectivity rules can also make up equality and inequality constraints where the former includes constraints of the form “choose exactly one” and the latter includes constraints of the form “choose at least one”. Continuous variables within superstructure optimisation formulations include state flowrates and compositions as well as intermediate state variables used in the objective function evaluation, whereas integer variables are used for the activation/deactivation of process units and interconnections.

#### 3.4.3 Process unit models

Once the superstructure has been postulated, its comprising process units and streams must be formulated mathematically such that they can be included in the optimisation problem. The choice of models depends on the complexity of the underlying processes being represented and the desired accuracy of the solution. As mentioned earlier, there exists different approaches to formulate mathematical models for embedding in the superstructure: direct incorporation of rigorous models, short-cut models, algorithmic function calls to process simulation software, or surrogate modelling.

Rigorous models of process unit transformations account for reaction kinetics and mass/heat transfer using fundamental equations, thereby offering the most accuracy in the final solution. For the application to wastewater systems, some mathematical models exist (such as ADM1 for anaerobic digestion (Batstone et al., 2002b), ASM1 for aerobic bioreactors, clarifier models (Otterpohl and Freund, 1992), etc.); however, many of these models, owing to the biological nature of the underlying processes, are highly dimensional and complex. Such models are also typically non-convex leading to issues guaranteeing global optimality. Additionally, rigorous models can become particularly cumbersome in integrated process design due to the combinatorial nature of superstructures (Alva-Argáez et al., 1998).

Process simulation software, embedding state-of-the-art rigorous models as well as algorithms to enable solution convergence for complex process networks, can therefore be used to evaluate input-output relationships from the underlying complex functions. However, this direct simulation-based optimisation approach has two problems. Firstly, the function calls to the process simulators can be computationally expensive as the simulator is allowed to converge on each evaluation leading to impractical CPU times. Secondly, the complex models within these process simulators are often implemented as “black boxes” with no available functional form or derivative information (Caballero and Grossmann, 2008), thereby introducing errors due to derivatives obtained by finite differences (Chen and Grossmann, 2017). Approximations are therefore often necessary to make superstructure optimisation problems tractable.

In order to harness the rigorous models within process simulation software, another approach uses the process simulators as a source of computer experiments which capture the underlying model input-output relationships of relevant variables. This high-fidelity data can then be used to fit tractable and/or reduced-order surrogate models to be used in surrogate-based optimisation (Boukouvala, Hasan, Floudas, 2017, Boukouvala, Ierapetritou, 2013, Bradley, Kim, Kilwein, Blakely, Eydenberg, Jalvin, Laird, Boukouvala, 2022, Caballero, Grossmann, 2008). The surrogate modelling methodology therefore has two important steps: generation of the input-output data from the process simulator and subsequent fitting of surrogate models.

Two different approaches also exist for connecting process unit models so as to represent multi-stage reaction pathways within the superstructure. The first approach formulates modular mathematical models for each individual process unit before splicing these models together within the optimisation formulation using logic constraints (Kim and Boukouvala, 2020). The second approach formulates superstructure-wide models mapping input-output variables for each possible pathway through the superstructure. The former results in a number of models equal to the number of process units incorporated in the superstructure, but challenges exist in splicing these models together, particularly for complex state definitions (e.g., for wastewater characterisations) which map the output of one task onto the inputs of another. Conversely, systems-wide models do not require splicing but the number of models required increases rapidly owing the combinatorial nature of the superstructure.

#### 3.4.4 Global optimisation of superstructures

The solution to superstructure optimisation problems determine the optimum configuration of technologies as well as optimised design and operating variables from within the postulated design space. To solve the optimisation problem, sophisticated algorithms developed over the last 60 years (Bixby and Rothberg, 2007) can be implemented, where the choice of solver depends largely on the type and complexity of the MIP problem. There are two broad categories of optimisation solvers, deterministic and meta-heuristic, where the former guarantee convergence to a global optimum for convex problems and the latter do not provide this guarantee in finite time (Chen and Grossmann, 2017).

For the case of MINLP problems, the two most popular deterministic global optimisation solvers are BARON (Sahinidis, 1996) and ANTIGONE (Misener and Floudas, 2014) which work by implementing convex relaxations and iteratively tightening these using branch-and-cut approaches. As an alternative to rigorous deterministic methods, non-rigorous meta-heuristic methods exploit cheap objective function evaluations and heuristic-based search algorithms to solve MINLP problems, in particular when derivatives are not available and finite-difference approximations are too expensive to perform (Regis and Shoemaker, 2005). Disadvantages of meta-heuristic methods are large numbers of function evaluations leading to long computational times required for convergence, and the algorithms search only for stationary points so finding the global optimum can involve lots of restarts as the algorithm gets stuck in local optima (Regis and Shoemaker, 2005). Examples of meta-heuristic optimisation methods include genetic algorithms (Deep, Singh, Kansal, Mohan, 2009, Goldberg, 1989) and simulated annealing (Kirkpatrick et al., 1983).

As mentioned in the introduction, the similar area of planetary boundary compatible power system design has been address using an optimisation-based model, such as by Algunaibet et al. (2019). Formulating the design problem as a linear program allows the optimum to be identified directly, without use of heuristic methods. The objective function used was minimisation of the sum of the PB transgression for each PB.

#### 3.4.5 Plant-wide modelling for wastewater treatment & resource recovery

Within WWTRR, plant-wide modelling can be used as a tool to assist in developing understanding of a system-level behaviour, particularly the interactions between different unit processes, allowing globally optimum solutions to be found (Mannina et al., 2016). If a reliable plant-wide model can be constructed, a variety of system proposals can be investigated at the design stage without time-consuming and expensive pilot systems (Kong, 2018). Alternatively, at the operations stage, experiments can be run using the model in order to de-risk experiments on the real-world system (Kong, 2018).

**Fig. 13:**
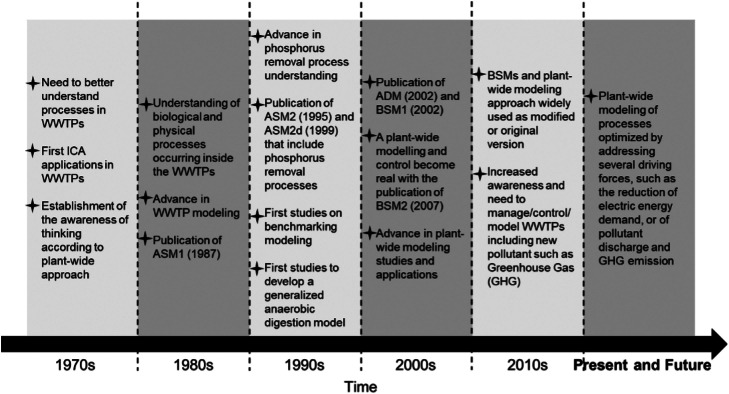
Milestones in the evolution of wastewater treatment plant modelling. Figure from Kong (2018), adapted from Mannina et al. (2016). WWTP – Wastewater treatment plant. ICA – Instrumentation, Control, and Automation. ASM – Activated Sludge Model. BSM – Benchmark Simulation Model.

Fig. 13 gives an overview of the evolution of wastewater treatment plant modelling. One of the drivers of the application of mathematical models to wastewater treatment processes was the introduction of instrumentation, control and automation to wastewater treatment plants, beginning in the 1970s, as this led to both improved process understanding and a greater need for models (Mannina et al., 2016). However, in the early years, research was stymied by different research groups using different, incompatible models of wastewater treatment. The publication of ASM1 (Activated Sludge Model 1) in 1987 (Henze et al., 1987) was intended to address this problem, by providing a common mathematical description of the activated sludge process. Since 1987, more complex models have been published as part of the ASM ’family’ of models, to reflect improved scientific understanding of activated sludge, such as the role of biological phosphorus removal, first incorporated into ASM2 (Gujer et al., 1995), and denitrifying polyphosphate-accumulating organisms, added in ASM2d (Henze et al., 1999).

Building on the ASM models, benchmark simulation models (BSMs) were also developed during the 2000s. The BSM1 model adds a secondary clarifier to a five-compartment activated sludge bioreactor described by ASM1, and includes representative influent data for dry, rainy and stormy periods (Copp, 2002). The BSM2 model builds on the BSM1 model, but with the addition of a primary clarifier, and anaerobic sludge digester to allow evaluation of control strategies at the plant-level (Jeppsson et al., 2007). These models were primarily intended to facilitate the evaluation and comparison of control strategies (Copp, 2002, Jeppsson, Pons, Nopens, Alex, Copp, Gernaey, Rosen, Steyer, Vanrolleghem, 2007). However, the adoption of BSM models for the evaluation of control strategies also demonstrated that numerical models of wastewater treatment plants could be used to predict operational performance, promoting the use of numerical models in process design as well as control design.

Use of models for WWTRRP synthesis has been explored within academic literature. Behera et al. (2020) presented a process synthesis tool for WWTRRP, used to perform a super-structure optimisation based on available technologies, selected constraints and the objective function of choice, along with three case studies demonstrating its use. However, the optimisation was limited to heat and energy recovery, focused on domestic, rather than industrial, wastewaters, and didn’t consider the potential for other resources such as nitrogen and phosphorus to be recovered. Bozkurt et al. (2016a) presented and applied a superstructure optimization framework for WWTP process selection to the retrofit of 2 domestic WWTP plants, with a minimum cost objective.

Another use of numerical WWTP models is for predicting the performance of existing facilities in changed conditions. Zhang et al. (2021) presented a machine-learning (random forest) based model for the prediction of WWTP energy consumption based on different effluent discharge standards. While the intention here wasn’t WWTRRP synthesis, a similar methodology could be used for predicting the energy consumption of a hypothetical plant - the authors note the speed, flexibility and robustness of the ML model relative to the first-principles models used in process simulators.

#### 3.4.6 Combining expert knowledge with ML tools

Integrating ML models with expert knowledge is a common goal in applied ML studies in different areas (von Rueden et al., 2021). In their large-scale review, von Rueden et al. (2021) identify use of algebraic equations, probabilistic relations and human feedback as the most widespread methods of addressing this goal. Expert insights can also be vital in shaping penalty functions used within ML models, such as by penalising non-physical results, and in defining an appropriate search space (Seidenberg et al., 2023). However, there are relatively few studies that combine expert knowledge with ML models within the domain of WWTRRP design and optimisation. Ho et al. (2021) used a fuzzy logic approach to assign weighting factors to different design criteria based on judgements from industrial domain experts, for design of a sustainable WWTP, with trade-offs between total investment cost, carbon footprint and plant footprint. A similar approach could be used within the PB or resource recovery framework.

In the broader area of process design, Seidenberg et al. (2023) combined expert knowledge and artificial intelligence using ontological knowledge graphs, hierarchical reinforcement learning agent, and an equation-oriented simulation environment to design a steam methane reforming process. This draws on an system of expert process engineering knowledge published previously (Morbach et al., 2007). The system of knowledge is used to interpret the decisions made by the reinforcement learning agent, which can then be tested in the simulation environment.

An alternative approach to process optimisation, for an established biomanufacturing process, is employed by Manapragada et al. (2023). Citing the high complexity, variability and uncertainty associated with pharmaceutical bioprocesses, considerations that equally apply to WWTRRP, they decide to pursue data-driven process optimisation by providing decision support tools domain experts, rather than full automation. The objective adopted was to optimise the feed strategy for a fed-batch culture, with a NN, trained on historic process data, suggesting feed rates and nutrient concentrations based on the rector state.

While any real application of ML tools for process synthesis and optimisation will involve some degree of expert judgement in employing the tool, and accepting the results, formal approaches to this integration are still in the process of maturing, with relatively few applications in process design, and fewer within WWTRRP design specifically. The examples discussed demonstrate the different approaches to combining expert judgment with AI. Employing and adapting these existing methods to WWTRRP, and developing new expert-ML integration approaches in WWTRRP could significantly ease the increasingly important WWTRRP design problem.

### 3.5 Black box optimisation

**Fig. 14:**
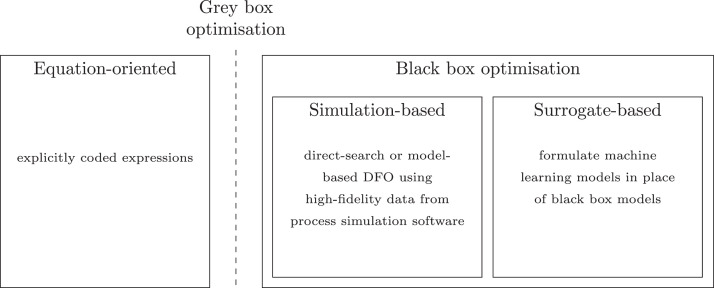
Equation-oriented and black box optimisation including simulation-based and surrogate-based optimisation. Grey box optimisation exists at the interface of equation-oriented and black box optimisation. DFO: derivative-free optimisation.

Unlike equation-oriented optimisation wherein the mathematical formulations of f and g (Equations 1a–1b) are explicitly known, BBO considers cases in which the mathematical formulations of f and g are unknown or not readily available (Fig. 14). At the intersection of equation-oriented optimisation and BBO, grey box optimisation considers cases where some mathematical formulations of f and g are readily available and some are unknown. Solving BBO problems, independent of derivative information for the unknown formulations, can be approached by DFO methods (Audet and Hare, 2017). A key challenge facing BBO is the accurate and tractable formulation of unknown f and g models within mathematical programming problems.

DFO methods can be categorised into direct-search methods and model-based methods. The former sequentially examines samples for improvements in optimally, whilst the latter involves approximating underlying functions (e.g., f replaced by $\hat{f}$) and guiding the search using surrogate models. Algorithms can be further categorised as local or global DFO methods where the former excel at refining current best solutions to obtain local optima, whilst the latter facilitate exploration for global solutions within the search space. Finally, stochastic DFO methods differ from deterministic algorithms due to the incorporation of random search steps. Rios and Sahinidis (Rios and Sahinidis, 2013) provide an extensive review of DFO algorithms and a comparison of available software implementations.

In BBO, simulation software embedding rigorous, complex models is interrogated for input-output data followed by DFO, wherein the derivatives of the underlying model are not directly used (Boukouvala, Misener, Floudas, 2016, Conn, Scheinberg, Vicente, 2009, Rios, Sahinidis, 2013). BBO methodologies can be categorised into three broad frameworks: sampling-based, surrogate-based, and stochastic or evolutionary methods (Kim and Boukouvala, 2020). Sampling-based methodologies interrogate the black box and then use direct search and bound tightening algorithms to choose subsequent samples that either refine the solution or explore other areas of the search space. In a similar way, stochastic or evolutionary methods sample a large population from the simulation and then use heuristics to update characteristics of the entire population towards optimality. Finally, surrogate-based optimisation involves fitting a surrogate (also known as meta- or reduced-order) model to input-output data sampled from the simulation, then performing optimisation on the surrogate. Catalysed by recent interests and developments in ML, surrogate-based optimisation has been applied across diverse fields (Boukouvala, Floudas, 2017, Hastie, Tibshirani, Friedman, 2009, Yondo, Andrés, Valero, 2018, Zhang, Han, Gao, Wang, 2019).

Simulation-based optimisation is a subset of BBO wherein state of the art process simulation software is harnessed as a source of rigorous process models (f and/or g) as well as algorithms to enable simulation convergence for complex process networks. Interfacing optimisation programs to process simulation software thereby enables direct evaluations and active ML using high-fidelity input-output data from underlying f and/or g models. Subsequently, the data can be used by DFO methods to guide direct-search optimisation algorithms (direct-search DFO) or to train surrogate models to provide representative formulations to embed within mathematical programming frameworks (model-based DFO). In the case of direct-search methods, convergence criteria can take numerous formulations: perturbation of the solution below some tolerance; change in the objective function value below some tolerance; change in the (global or local) surrogate model accuracy below some tolerance; or some combination of these. Amaran et al. (Amaran et al., 2016) provide a comprehensive review of simulation-based DFO algorithms and applications.

A further subset of BBO is surrogate-based optimisation wherein representative yet tractable formulations of unknown f and g models are incorporated within mathematical programming frameworks (Alarie et al., 2021). These mathematical reformulations are referred to as surrogate models and are fit to training data in a supervised ML approach. The training data for surrogate models can come from various sources such as data lakes including sensor data, physical laboratory experiments, or computer experiments using process simulation software to enable the rigorous black box models to be exploited. However, a challenge pertains to the quality of these different data sources; sensor data can be noisy with large uncertainties from the practical operating environment, physical experiments can be unrepresentative of different operational scales, and computer experiments are evaluated through a layer of abstraction due to the underlying black box models.

Surrogate-based optimisation has gained popularity due to the increase in data availability with which to fit surrogate models, and improvements in global optimisation (Alarie et al., 2021). In addition to providing mathematical formulations of the underlying models, surrogate models also address the computational intractability of incorporating rigorous models directly within equation-oriented decision-making frameworks. Surrogate models reduce the computational cost of evaluating expensive model relationships via a reduction in model complexity and/or dimensionality. As such, surrogate modelling is widely used for predictive modelling and feasibility analysis as well as mathematical optimisation (Bhosekar and Ierapetritou, 2018a). Two main emerging methods concern the use of global surrogates versus iteratively updated local surrogates. The former involves fitting a surrogate model representative of the entire design space and performing optimisation to determine the globally optimal solution for this one representation of the underlying data. The latter follows a more Bayesian approach of iteratively updating the surrogate model based on consecutive optimisation solutions highlighting regions for more exploration or exploitation (Bhosekar and Ierapetritou, 2018a).

By formulating surrogate models within optimisation problems, their predictive power can be harnessed in solving black box decision-making problems (Boukouvala, Hasan, Floudas, 2017, Boukouvala, Misener, Floudas, 2016). However, a primary challenge lies in writing surrogate model formulations that are tractable within an optimisation problem. That is, translating a predictive formulation of an ML model into a formulation that is compatible with optimisation solver software. Recent literature has also addressed this challenge (Ceccon, Jalving, Haddad, Thebelt, Tsay, Laird, Misener, Maragno, Wiberg, Bertsimas, Birbil, Hertog, Fajemisin, Schweidtmann, Mitsos, 2019), harnessing recent advances and popularity of optimisation modelling language Pyomo (Bynum et al., 2021) and Python-based ML packages, e.g., Scikit-learn (Pedregosa et al., 2011) and PyTorch (Paszke et al., 2019). Specifically, these works utilise the object-orientated paradigm to enable the abstraction of surrogate model formulations into independently contained objects which can then be plugged into larger optimisation problems where required. For example, optimisation formulations for neural networks (NNs) exist widely in the literature, owing to both to their popularity as ML models and their highly customisable yet manageable structure (Grimstad, Andersson, 2019, Schweidtmann, Mitsos, 2019, Tjeng, Xiao, Tedrake). The weights matrices and bias vectors are optimised during model training and then fixed as optimisation parameters. Since the input to the activation function is a linear weighted sum, it is possible to formulate MILP problems depending on the activation functions used (Grimstad, Andersson, 2019, Tjeng, Xiao, Tedrake).

Caballero and Grossmann (Caballero and Grossmann, 2008) present a model-based DFO algorithm using GPs as surrogate models. The authors develop a bounds refinement algorithm to enable convergence upon surrogate-based optima with high confidence of accurate representation of the underlying model. Henao and Maravelias (Henao and Maravelias, 2011) use NNs to represent multi-variable mappings alongside explicit constraints within a grey box optimisation framework. In both cases, data for training surrogate models were obtained by using static sampling strategies to generate input samples and evaluating the corresponding outputs by interfacing Matlab with process simulation software. The former formulated NLP problems within the TOMLAB optimisation environment in Matlab (Holmström and Edvall, 2004) and solved these using SNOPT (Gill et al., 2002). The latter introduced discrete variables to represent a superstructure for optimising process synthesis, and solved the resulting MINLP problem with GAMS (Bussieck and Meeraus, 2004) and the DICOPT solver (Grossmann et al., 2002).

Boukouvala and Floudas (Boukouvala and Floudas, 2017) introduced a DFO framework for constrained grey box problems embedding sampling, bounds refinement, variable selection, surrogate modelling, and global optimisation. A key feature of their work was the incorporation of multiple surrogate functions (linear, quadratic, signomial, radial basis functions (RBFs), and kriging), from which the choice of model to be used was optimised. Beykal et al. (Beykal et al., 2020) incorporated support vector machines (SVMs) in within the DFO framework as supervised classification surrogate models to map the region of numerical infeasibility arising from noisy simulations. Both applications formulated NLP problems which were solved by tuning the solver parameters of ANTIGONE (Misener and Floudas, 2014) to enable local/global solutions.

Despite the rise in popularity of surrogate-based optimisation, most of these applications implement NLP wherein optimisation is performed on a continuous search space. There has been relatively little research on the applications of surrogate-based optimisation to MINLP. The challenges facing black box MINLP (bb-MINLP) optimisation frameworks are numerous: first, obtaining a representative yet tractable sample set that exists within a discontinuous search space; second, fitting a surrogate model to response surfaces involving continuous and integer decisions; third, formulating multiple continuous surrogates then patching them together at discontinuities can result in complex and less tractable optimisation formulations, particularly as the number of discrete decisions increases (Kim and Boukouvala, 2020).

Despite recent advances in simulation-based DFO, there remains many open challenges constraining the development and deployment of such algorithms and decision-support tools to real-world applications. A key challenge results from fragmented research approaches developing boutique DFO methods for specific applications, with no general one-size-fits-all solution and difficulty comparing methods to determine the best method for a given application. This challenge is compounded by the breadth of DFO applications and variations, including accounting for noisy outputs from stochastic simulations, optimising constrained BBO problems, and optimising both continuous and discrete variables. A second challenge derived from the fragmented research approach is the discontinuation of development after successful application. Recent research advances should be incorporated within existing DFO approaches as well as within commercial simulation software to enable simulation optimisation without necessitating programming experience and interfacing to a programming language. Finally, guaranteeing global solutions within reasonable computational time, particularly for highly dimensional and complex BBO problems, is an ongoing challenge in the optimisation community.

#### 3.5.1 Black box optimisation in wastewater treatment & resource recovery

Studies looking at the application of black box optimisation to WWWTRR are relatively few. Moreover most works focus on treatment of the wastewater, and neglect resource recovery as an objective. For a more comprehensive review of artificial intelligence techniques, which are related to black box optimisation, in wastewater treatment, albeit limited to electrochemical processes, see Shirkoohi et al. (2022).

One approach is to model high-quality process simulators as black boxes, with BBO then used to identify improved designs or operating regimes. This approach was taken by Gradano and Le Roux (Gradano and Le Roux, 2012) and Foschi et al. (Foschi et al., 2021). Gradano and Le Roux (Gradano and Le Roux, 2012) compared the performance of a polynomial black box model and a neural-network model as surrogate models for a wastewater treatment process composed of a cooling-tower and steam stripper, simulated using MATLAB and Aspen Plus. The optimisation problem was formulated as an MINPL. The results showed that both the approaches are computationally tractable with a similar accuracy, suggesting a role for surrogate models of these types in the design of realistic water treatment plants. Foschi et al. (Foschi et al., 2021) also used a neural-network approach, for process control of UV wastewater disinfection, as a surrogate for a computational fluid dynamics model of the process which required lengthy computations and was therefore unsuitable for control applications. A pre-print article by two of the co-authors of this paper investigated both gaussian processes and neural networks as surrogates for WWTRR processes (Durkin and Guo, 2023), applying the modelling tools developed to energy and nutrient recovery from brewery wastewater, and demonstrating the ability of the modelling methodology to address process synthesis for resource recovery whilst also accounting for uncertainties inherent to wastewater systems (Durkin and Guo, 2023).

A common approach is to use response surface methodology to characterise the process of interest (Mook, Aroua, Szlachta, Lee, 2017, Thirugnanasambandham, 2021). These methods have been used by Thirugnanasambandham to investigate treatment of food wastewater using aerobic mixed microbial culture and electroflocculation (Thirugnanasambandham, 2021), and by Mook et al. to explore electrocoagulation treatment of textile wastewater (Mook et al., 2017). Another data-based modelling technique is support vector machines (SVMs). Yang et al. looked at SVM as a black-box modeling technique to model nitrogen removal in biological wastewater treatment (Yang et al., 2006). More specifically, a least squares - support vector machine (LS-SVM) with nonlinear autoregressive network with exogenous inputs (NARX) was used, and found to have good generalization performance. Nezungai and Majozi were more critical of black box modelling, however, showing the black box approach can lead to inaccuracies of up to 85% in the costing of regeneration units in multi-contaminant electrodialysis (Nezungai and Majozi, 2016).

#### 3.5.2 Object-Oriented programming in derivative-free optimisation

Some of the problems associated with fragmented DFO development can be addressed by harnessing object-orientated programming (OOP) to provide general modelling toolboxes. Such approaches provide flexible foundation models which can be configured and adapted to specific applications. The flexibility of developed DFO methods is thereby increased by addressing the various modelling challenges at the object-level as opposed to the algorithm-level. OOP further lends itself to open-source development and the incorporation of research advances within new or updated modelling objects which can improve the performance of existing DFO configurations. Finally, modelling objects can also be incorporated within tailored DFO implementations in a broad range of commercial simulation software. OOP for DFO also enables the dissociation between mathematical programming formulations and optimisation solvers, enabling local or global rigorous gradient-based solvers or stochastic metaheuristic solvers to be adopted as required.

A number of object-orientated surrogate modelling and DFO toolboxes have been developed to date, including the SUMO toolbox available in Matlab (Gorissen et al., 2010) and the surrogate modelling toolbox (SMT) available in Python (Bouhlel et al., 2019). The former enables numerous surrogate models to be trained and updated using adaptive sampling methods, with a primary focus on enabling more computationally tractable models for predictive purposes, whilst the latter enables static sampling strategy implementations and different surrogate model formulations with a focus on derivatives for use in gradient-based optimisation. Cozad et al. (Cozad et al., 2014) developed a machine learning software able to interface with many black box simulation codes and construct surrogate models from a selection of available basis functions, as well as adaptive sampling implementations, within a no-code interface. The optimisation and machine learning toolkit (OMLT (Ceccon et al., 2022)) is a recently developed open-source python package for formulating neural network (NN) and gradient-boosted tree surrogate models within larger optimisation problems. Audet et al. (Audet et al., 2021) recently updated their popular implementation of the mesh adaptive direct-search algorithm for DFO to a new object-orientated architecture to facilitate greater flexibility.

(Durkin and Guo, 2023) used an objected oriented approach to surrogate modelling and DFO. The objected-oriented modelling tools developed to facilitate application of derivative-free methods to black box optimisation problems are available on GitHub(Durkin, 2022).

### 3.6 Summary of the state of the art

The wastewater sector is undergoing a paradigm shift from wastewater treatment towards more sustainable resource recovery (van Loosdrecht and Brdjanovic, 2014). Such a shift is part of a larger transition towards a circular economy needed to alleviate the environmental concerns associated with unsustainable resource extraction and wastefulness (Lieder and Rashid, 2016). Specifically, the agri-food system needs to undergo a sustainable transition owing to its large contribution to the transgression of the safe operating limits for climate change, nitrogen fixation, phosphorus loading, freshwater use, and land use (Campbell et al., 2017). In this regard, resource recovery from FPWW provides an environmentally beneficial solution, yet remains relatively unexplored (Durkin et al., 2022b). However, FPWW remains a large application domain so microbial food and drink effluents, produced in controlled environments, are highlighted as specific FPWWs providing synergistic solutions to reduce the environmental impacts of traditional agricultural, specifically animal-dependent, production processes (Banks et al., 2022).

Despite wide research on technology development for resource recovery from FPWW, there exists numerous barriers constraining their successful deployment to industry (Costa et al., 2022). For industry to commit to expensive pilot studies, the research gap surrounding early-stage process synthesis and design methods to address the challenges of resource recovery from FPWW must be addressed (Diaz-Elsayed, Rezaei, Guo, Mohebbi, Zhang, 2019, Yadav, Mishra, Ghosh, Sindhu, Vinayak, Pugazhendhi, 2021). Research challenges include: holistic decision-making accounting for multiple competing objectives (Villarín and Merel, 2020); the complexity and uncertainty of wastewater characterisation and plant operations (Tóth et al., 2022); mathematical modelling of resource recovery processes in the context of wastewater systems (Solon et al., 2019); integrative process design to select from multiple technologies and multiple resources to be recovered (Guerra-Rodríguez et al., 2020); and how to implement resource recovery within practical operating environments whilst ensuring feasible process operation at an industrial scale (Durkin et al., 2022b).

PSE is well poised to harness recent developments in ML to develop decision-making frameworks to optimise process systems for sustainable resource recovery from FPWW (Sanford et al., 2016). Despite PSE applications to the valorisation of organic solid waste in biorefineries, applications to FPWW are relatively lacking (Leong et al., 2021). Previous decades have resulted in the development of first-principles model for wastewater treatment processes and the incorporation of these models within commercial process simulators. However, optimising process systems using these process simulators necessitates expert domain knowledge and sequential process synthesis which can result in sub-optimal process systems (Westerberg, 2004). PSE-derived superstructure optimisation methodology thereby enables systems-wide optimisation which has been applied to wastewater treatment (Bozkurt, van Loosdrecht, Gernaey, Sin, 2016, Castillo, Cheali, Gómez, Comas, Poch, Sin, 2016) whilst applications to resource recovery from FPWW remain unexplored. Additionally, superstructure optimisation itself poses the following research challenges: postulation of the design space; computational tractability of modelling and optimising integrative superstructures (Alva-Argáez, Kokossis, Smith, 1998, Gong, You, 2015); methods to hedge against process and model uncertainties (Gong and You, 2015a); and solving the optimisation problem for the holistically global optimum and the corresponding definition of optimality (Chen, Grossmann, 2017, Gong, You, 2015, Gong, You, 2015).

The optimisation of superstructures for wastewater treatment have utilised short-cut models to enable representation of rigorous process models within the decision-making process (Bozkurt, Quaglia, Gernaey, Sin, 2015, Puchongkawarin, Gomez-Mont, Stuckey, Chachuat, 2015). However, such short-cut approaches, whilst ensuring computational tractability, result in considerable loss of model accuracy. As such, there exists a research gap to harness high-fidelity data from underlying rigorous models within process simulation software. Specifically, simulation-based optimisation of resource recovery from FPWW systems is possible via DFO approaches including simulation-based or surrogate-based optimisation (Bradley et al., 2022). Simulation-based optimisation can be approached via direct-search DFO wherein high-fidelity data is used to guide the optimisation search. However, the requirement for process models enabling increased interpretablity and trust in modelling solutions necessitates a large number of simulation evaluations resulting in excessively high computational costs. Additionally, the underlying code within process simulation software is often embedded as black box models where functional and derivative information is unavailable to the user (Amaran et al., 2016). On the other hand, surrogate-based optimisation necessitates an ML approach to model training on high-fidelity data which is constrained by a dissociation between traditional engineering specialists and computer science knowledge (Corominas, Garrido-Baserba, Villez, Olsson, Cortés, Poch, 2018, Dobbelaere, Plehiers, Van de Vijver, Stevens, Van Geem, 2021, Hadjimichael, Comas, Corominas, 2016, Himmelblau, 2008, Newhart, Holloway, Hering, Cath, 2019).

Developments in PSE must therefore bridge the gap to the wastewater treatment sector to assist in the paradigm shift to sustainable resource recovery (Pistikopoulos et al., 2021). To assist in bridging this gap, decision-making frameworks should be developed as general modelling toolboxes so as to enable configuration and adaptation to specific applications. Additionally, the developed toolbox should provide sufficient tools to assist the full workflow for DFO-informed process synthesis for resource recovery from FPWW with a focus on simulation-based optimisation enabling state of the art simulations embedding domain specific knowledge to be harnessed as data-source for data-driven optimisation of a wide range of different applications, including: high-fidelity data sampling and processing from wastewater simulation software; surrogate modelling and validation including classification models to handle infeasible process designs; tractable mathematical programming formulations for classification and regression models as optimisation constraints; integrated adaptive sampling objects harnessing mathematical programming formulations for deterministic optimisation of subsequent computer experiments; and DFO solution algorithms including direct-search and model-based approaches.

## 4 Final remarks & recommendations for future work

### 4.1 Final remarks

Meeting the growing demands of an increasing global population whilst operating within established environmental planetary boundaries (PBs) is an important and timely challenge. The linear take-make-waste economy and inefficient modes of agricultural production were highlighted as primary drivers of the PB transgressions, with innovative disruption needed to bring about more sustainable practices. The recovery of resources from food and beverage processing wastewaters (FPWW) has been posed as a synergistic solution to alleviate the associated environmental issues. However, the successful deployment of resource recovery processes requires a “systems thinking” approach to determine holistic solutions, together with a willingness to move away from established processes where these are wasteful or unsustainable. Process systems engineering (PSE) provides a scientific discipline and computational methodologies, particularly with the recent gains in data-driven artificial intelligence (AI) and machine learning (ML) capabilities, to address the challenge of synthesising resource recovery systems for wastewater (Pistikopoulos et al., 2021), while the life-cycle assessment (LCA) and the planetary boundary (PB) frameworks allow the sustainability of the different solutions to be assessed on an absolute, as well as relative, basis (Algunaibet et al., 2019).

Since the coinage of Moore’s Law in 1975 (Moore, 2006), the realisation of the predicted exponential increases in computational speed and capability have been revolutionary. The PSE community has often been among the first to benefit from these advances, employing improved computational capacity to accelerate models, analyse ever larger datasets, increase the scale and granularity of optimisation problems, and apply AI and ML methods to PSE problems. PSE is therefore well positioned to build on the developments presented in this work to address the technical challenges surrounding greater resource recovery from wastewater, as part of the move towards a circular economy (Pistikopoulos et al., 2021).

In addition to technical challenges such as process synthesis and design and optimisation of individual wastewater treatment reactor types, the transition towards widespread resource recovery from wastewater will also involve addressing economic, political, regulatory and public communication challenges, with a significant role for both consumers and state, corporate and third-sector actors. While the degree of public control of municipal wastewater treatment plants varies by jurisdiction, in many countries industrial sites, such as food and drink manufacturing sites, operate their own sewage treatment works. The operation of these sites will be guided by commercial considerations, as well as legal and environmental ones. Government measures to encourage resource recovery include tightening discharge limits or charging for nutrient discharge, and incentivising resource recovery, through subsidies or price guarantees for recovered resources. As well as supply, demand for recovered resources is needed for a healthy market. While certain recovered products, such as bio-gas, are fairly well-established and standardised (European Biogas Association, 2023, ISO, 2018), for other recovered resources such as other bio-fuels, nitrogen/phosphorus-rich fertilisers, or clean water, careful management of consumer perception and sensible standards are needed to foster uptake.

### 4.2 Recommendations for future work

There exists multiple directions for future PSE research in the field of surrogate-based optimisation of wastewater treatment and resource recovery (WWTRR). On a more fundamental level, there is work to be done in increasing the transparency and interpretability of the machine learning models used within optimisation frameworks, and exploring the use of different surrogate model structures that could allow more accurate modelling of non-linear process systems. Examples of surrogate model structures that have been highlighted include deep learning with recurrent neural networks, support vector machines, random forests, and decision trees. The benefits of alternative model structures can be varied: for example, the aforementioned decision trees provide predictions along with their reasoning which can be interpreted by humans. Model interpretability helps human operators and decision makers trust surrogate models, making deployment more likely.

Another area of fundamental research is how to best combine ML models with expert domain knowledge. Practical applications of ML often need to be combined with expert knowledge to exploit large volumes of data while retaining model transparency, interpretability, and extrapolation capabilities (Schneider et al., 2022). This can be achieved by combining ML surrogate models with well-established mechanistic models and expert domain knowledge to assist in gaining the trust of practical operational engineers without ML modelling experience. Such hybrid models should help with the interpretability of black box simulators, increasing the transparency of black box optimisation. However, the application of such hybrid intelligence models to the challenge of resource recovery from wastewater remains relatively unexplored.

In terms of applications research, artificial intelligence could be used to predict variations in quantities and qualities of waste feedstocks (Zhu et al., 2022). By analysing large volumes of data, machine learning models can correlate waste characterisation data with environmental variables to enable future projections of waste production and compositions (Robles et al., 2021). Artificial intelligence thereby enables temporal and spatial modelling of resources available for recovery from waste and allocation to resource recovery processes, leading to better design, control and operation decisions in WWTRRPs.

PSE optimisation and process synthesis tools could be combined with the PB-LCA framework to design absolutely sustainable WWTRR plants. While in high-income countries, with 70% of wastewater already treated (Dutta et al., 2021), resource recovery facilities will need to be designed to complement existing infrastructure, in low and middle income countries, where most wastewater is not yet treated (Dutta et al., 2021), there is an even greater opportunity to exploit the resources present in FPWW and other wastewater, as WWTRR facilites can be designed from scratch, and so co-optimised for resource recovery (maximising the value returned to supply chains (Durkin et al., 2022b)) and effluent quality. While some work on the absolute sustainability of WWTRR has already been done (Ryberg et al., 2020), PB-LCA needs to move from being a process analysis tool to a widespread process synthesis tool, if the ideals of circular economy and sustainable biorefineries are to be realised. This kind of methodology has already been employed within PSE to design electricity systems (Algunaibet et al., 2019). However, there remains a need for integrated WWTRR synthesis studies at plant, city and national scales; due to the inherent complexities and uncertainties of wastewater, surrogate-based optimisation may need to be adopted for robust results to be obtained.

## Funding

This research did not receive any specific grant from funding agencies in the public, commercial, or not-for-profit sectors.

## CRediT authorship contribution statement

**Alex Durkin:** Writing – review & editing, Visualization. **Tom Vinestock:** Project administration, Writing – review & editing. **Miao Guo:** Supervision, Conceptualization.

## Declaration of competing interest

The authors declare that they have no known financial interests or personal relationships that could have influenced or could appear to influence the work presented in this article.
